# Supplementary material for: A hybrid gravity and route choice model to assess vector traffic in large-scale road networks
Source: R Soc Open Sci. 2020 May 20;7(5):191858. doi: 10.1098/rsos.191858 (PMC7277278; doi:10.1098/rsos.191858)
Supplement: Supplementary Appendices for "A Hybrid Gravity and Route Choice Model to Assess Vector Traffic in Large-Scale Road Networks" [file rsos191858supp1.pdf]

ROYAL SOCIETY OPEN SCIENCE

rsos.royalsocietypublishing.org

Research

Supplementary appendices for published article

Author for correspondence:
Samuel M. Fischer
e-mail: samuel.fischer@ualberta.ca

Supplementary Appendices for "A Hybrid Gravity and Route Choice Model to Assess Vector Traffic in Large-Scale Road Networks"

S. M. Fischer[1], M. Beck[2], L.-M. Herborg[3] and M. A. Lewis[1,4]

[1]Department of Mathematical and Statistical Sciences, University of Alberta, Edmonton, AB.
[2]BC Ministry of Environment and Climate Change Strategy, Conservation Science Section, Victoria, BC.
[3]Fisheries and Oceans Canada, Institute of Ocean Sciences, Sidney, BC.
[4]Department of Biological Sciences, University of Alberta, Edmonton, AB.

THE ROYAL SOCIETY PUBLISHING

© 2020 The Authors. Published by the Royal Society under the terms of the Creative Commons Attribution License http://creativecommons.org/licenses/by/4.0/, which permits unrestricted use, provided the original author and source are credited.

A. Modelling assumptions

Below we provide a comprehensive list of our modelling assumptions.

(i) For each time unit, the number of travelling agents N_{ij} is given by a stochastic gravity model.
(ii) Each time unit, the number N_{ij} is drawn from a negative binomial distribution. Thereby, the numbers N_{ij} and N_{kl} for origin-destination pairs $(k, l) \neq (i, j)$ are independent of each other and of the past.
(iii) The distribution of N_{ij} is independent of the spatial scale at which we consider the system.
(iv) Agents choose their routes randomly and independently of each other.
(v) Most agents drive along a set of "admissible" routes. This route set should encompass all "major alternatives" that agents choose from.
(vi) A route is admissible if it does not contain local detours and is not much longer than the shortest route from the origin to the destination.
(vii) The probability to choose an admissible route is inverse proportional to a power of its length.
(viii) All agents who are not driving along an admissible route can be observed everywhere in the route network with the same probability.
(ix) Agents choose randomly the time of day when they pass a location on their route.
(x) The distribution of the time of day when an agent passes a certain location is independent of the location, the origin and destination of the agent, earlier time choices of the agent, and other agents' timing.
(xi) The temporal pattern determining when agents pass a survey location is a von Mises distribution.
(xii) Agents choose randomly and independently of each other whether or not they participate in the survey.
(xiii) The compliance rate is independent of the respective agents, the time when they pass the survey location, and the location of the survey.

B. Scale-invariant count distributions

A desirable property of spatial models is scale invariance. In the case of gravity models this means that the distribution of the number of trips starting or arriving at a region i should not change if we increase the spatial resolution and considered subregions i_1 and i_2 instead of i. That is, we require $N_{i_1 j} + N_{i_2 j} \overset{d}{=} N_{ij}$. See figure A1 for a depiction of the considered scenario.

If the agent counts in the subregions are independent of each other, Poisson random variables satisfy this condition always. However, independent negative binomial random variables satisfy this property only if they have the same mean to variance ratio, $p = \frac{\mu}{\sigma^2}$. This ratio measures the level of overdispersion of the distribution.

Following the claim that the gravity model is scale invariant and assuming that the agent counts in subregions are independent, we have to choose the mean to variance ratio p independently of origins and destinations. Hence, it makes sense to parameterize the negative binomial distribution in terms of the mean μ and the parameter p. Then, the probability mass function of N_{ij} reads

$$\mathbb{P}(N_{ij} = n) = \binom{n + r_{ij} - 1}{n} p^{r_{ij}} (1 - p)^n \tag{A1}$$

with $r_{ij} := \frac{p}{1-p} \mu_{ij}$.

Note that scale invariant distributions are also invariant against how locations are pooled together. Since large regions can be split in smaller regions without changing the cumulative distribution of the count data, the same applies also when smaller regions are connected to larger regions. Consequently, origin and destination regions can be chosen based on practical considerations without the risk of introducing a bias.

The negative binomial distribution can be understood as a Poisson distribution with a gamma distributed rate. That is, we could write

$$N_{ij} \sim Poisson\left(\mu_{ij} \lambda\right),$$

whereby λ is a gamma distributed random variable with mean 1. The random rate λ models that all agents' travel decisions may depend on common unknown factors, such as weather. If we hold the mean to variance ratio p constant, this implies that the variance of the rate $\mathbb{V}(\lambda) = c\mu_{ij}^{-1}$ is inverse proportional to

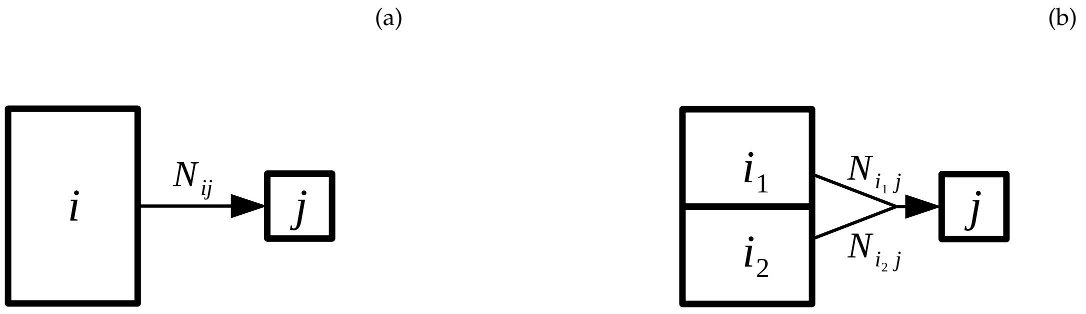

Figure A1: Scale invariance property. The total flow N_{ij} from a source i to a sink j (Panel a) shall not change if we split the source region into two subregions i_1 to i_2 and consider the two flows $N_{i_1 j}$ and $N_{i_2 j}$ (Panel b). Thus, $N_{i_1 j} + N_{i_2 j}$ has the same distribution as N_{ij}.

the mean number of travelling agents μ_{ij} with some proportionality constant c. This can be interpreted as a phenomenological model for mechanisms that reduce the variance of travelling agents at highly frequented destinations, where limitations of accommodations and other facilities may play a major role in reducing the variance of the agent inflow. If the model did not account for these factors, the model might predict an exceeding variance for count data from highly frequented locations.

C. Details of the data

This appendix contains details of the data used in the application section of this study.

(a) Data sources

The data sets that were compiled to be used in this study are available as electronic supplementary material. The sources of the original data used to generate the compiled data sets are displayed in Table A1.

(b) Variable spatial resolution

We used data with variable spatial resolution. Often it is hard to find or collect data with high spatial resolution. Similarly, incorporating high-resolution data in models can come with considerable computational challenges. However, typically, a high spatial resolution is only required in certain areas of interest. Consequently, it is advisable to use data with a resolution that is high in the area of interest and low elsewhere.

Following this principle, we split the province Alberta into three parts (north / Grande Prairie, middle / Edmonton, south / Calgary) and the state Washington into an eastern (Seattle) and a western (Spokane) part to get a better spatial resolution of the boater origins close to BC. Furthermore, we used a detailed road data set for BC and a sparse data set for the rest of Canada and the USA, because all survey locations and all roads of management interest were located in BC. If roads outside of BC were of management interest, a correspondingly adjusted road network would be required. The sparse road network we used contained highways only. In total, our road network consisted of 1.4 million vertices and 1.6 million edges.

(c) Data accuracy

In this section, we discuss the accuracy of the data we used.

(i) Survey data

The destinations of some surveyed boaters were not perfectly clear. The surveyed boaters were asked for their destination waterbodies and close-by cities. As not all lakes in BC have unique names and cities are rare in some regions of BC, we had to use common sense to deduce which lakes boaters went to, when the

Data	Source	URL
Boater Survey Data	BC Ministry of Environment	www2.gov.bc.ca/gov/content/invasive-mussels
Base GIS Data (e.g. lake data, borders)	BC Ministry of Environment	catalogue.data.gov.bc.ca/dataset
Road network	BC Digital Road Atlas	www2.gov.bc.ca/gov/content/data/geographic-data-services/topographic-data/roads
Angler Count Data Canada	Department of Fisheries and Oceans Canada	www.dfo-mpo.gc.ca/stats/rec/can/2010/section4-eng.htm
Angler Count Data USA	American Sportfishing Association	asafishing.org/uploads/2011_ASASportfishing_in_America_Report_January_2013.pdf
Population Data Canada	Statistics Canada	www.statcan.gc.ca
Population Data USA	U.S. Census Bureau	www.census.gov
Locations of Cities	Open Street Map	www.openstreetmap.org
Facilities (public toilets, tourist information, viewpoints, parks, attractions, and picnic sites)		
Campgrounds	USCAmpgrounds	www.uscampgrounds.info
	British Columbia Lodging and Campgrounds Association	www.campingrvbc.com/camping
Marinas	Manual web search for marinas in BC	–

Table A1: Data sources.

destinations were ambiguous. Thereby, we took into account the properties (size and available facilities) of the potential destination lakes and considered where the boaters were surveyed. As the data were unambiguous for highly frequented lakes, only a small fraction of the data were affected by the cleaning step. Nonetheless, for the lakes that we split due to their large size (see section (b)), some boater destinations may have been misclassified. Though these errors may result in skewed estimates of how many boaters use which section of a large lake, the errors should not have a major effect on the arrival estimates for the complete lakes. Clearly inconsistent or incomplete data were used only to determine the rate at which boaters provide trustworthy and complete data.

While erroneous and ambiguous data could be reduced by providing agents with a comprehensive list of possible destination locations, a second problem arises if surveys are conducted close to destination regions with multiple access points. A considerable number of agents accessing these recipient regions

may not pass the survey location if they are using other access points. Furthermore, the route choice model will be imprecise close to destination points unless they are known exactly, which is often not the case. Consequently, data collection in direct proximity of destinations with multiple access routes can yield unreliable results. This issue may also have affected our study.

(ii) Covariate data

As we collected the covariate data from external sources, we do not have specific insights on their accuracy. Note, however, that the angler data we used were collected by different agencies in Canada and the USA. This can lead to a bias if the classification of anglers is different in the two countries. We sought to reduce the potential resulting error by including the nation of source jurisdictions in the model.

D. Details of the model fit

In this Appendix, we provide details of how to fit the four submodels of the hybrid model and compute the likelihood functions efficiently. Furthermore, we outline the likelihood maximization procedure. Though we describe all important conceptual steps, we do not provide implementation details.

To make our explanations more understandable, we choose a specific time unit for this appendix. This contrasts with the main text, where we have have formulated our model in terms of a general time unit and left it up to the modeller to decide whether it is appropriate to model traffic as a repetitive process running in daily, weekly, or other cycles. Though we choose "days" as our time unit for this appendix, all the described methods apply without further limitations if a different time unit is used instead of days.

Slight adjustments to the presented equations may be necessary if multiple survey shifts are conducted during one time interval. In this appendix, we assume that at most one survey shift is conducted at a location per day. If it is possible that multiple, disjoint survey shifts are conducted in a time interval, the notion of "survey time interval" has to be replaced by "survey time set", and the computation of probabilities has to be adjusted accordingly. However, these adjustments concern only simple probability calculations and should be clear from the context.

(a) Fitting the compliance model

No sophisticated techniques are required to determine the compliance rate. To determine the participation rate, we simply determine the number of surveyed agents and divide it by the total number of agents passing our survey location. However, as it is typically impossible to know origin and destination or other properties of bypassing agents, the number of surveyed boaters should not be filtered by origins and destination or any other characteristics. Hence, it is important to record the compliance of agents that may not be of interest, unless these agents can be clearly distinguished from agents of interest without surveying them.

We proceed similarly to determine the proportion of agents that provide consistent and complete data. However, the origins of agents can often be determined easily based on license plate information once the agents have stopped for the survey. Hence, the complete data rate can be determined focusing on the agents of interest only.

(b) Fitting the temporal pattern model

The temporal pattern model describes the distribution of traffic over the day. When we fit this model, we have to recall that our sampling effort is not uniformly distributed over all day times. Therefore, we have to fit the model using the conditional likelihood.

Let T_i be the random variable describing when the i-th agent passes a survey location, and let $\left[t_i^{\text{start}}, t_i^{\text{end}}\right]$ be the time interval of the survey shift in which agent i was observed. As we can only observe agents who pass our location while we conduct the survey, $T_i \in \left[t_i^{\text{start}}, t_i^{\text{end}}\right]$ must hold for all agents i in our data set. Consequently, if f_{Time} is the probability density function of the temporal pattern model and F_{Time} the

respective cumulative density function, the likelihood function for our temporal pattern model reads

$$\begin{aligned} L_{\text{Time}}(\theta_{\text{Time}}) &= \prod_i f_{\text{Time}}\left(t_i^{\text{obs}}|\theta_{\text{Time}},\, t_i^{\text{obs}} \in \left[t_i^{\text{start}}, t_i^{\text{end}}\right]\right) \\ &= \prod_i \frac{f_{\text{Time}}\left(t_i^{\text{obs}}|\theta_{\text{Time}}\right)}{F_{\text{Time}}\left(t_i^{\text{end}}|\theta_{\text{Time}}\right) - F_{\text{Time}}\left(t_i^{\text{start}}|\theta_{\text{Time}}\right)}. \end{aligned} \tag{A1}$$

Here, t_i^{obs} is the time when the i-th agent has been observed, and θ_{Time} is the parameter vector for the temporal pattern.

Since both f_{Time} and F_{Time} are usually easy to evaluate and the computational complexity is independent of the system size and linear in the number of surveyed agents, no sophisticated algorithms are required to evaluate and maximize the likelihood.

(c) Fitting the route choice model

In this section, we provide instructions on how to fit the route choice model. We start by discussing conceptual details before we show how to evaluate the likelihood function efficiently by computing and reusing partial results.

We maximize the likelihood of the route choice model in a repeated two step procedure: first, we compute the set of admissible routes that most agents choose from. Then we fit the submodel that assigns the admissible routes with probabilities. We repeat these steps with different route admissibility parameters until a model is identified that maximizes the likelihood approximately.

The need for the two step procedure comes from our distinction between admissible and inadmissible routes. Whether or not a route is classified as admissible is a yes/no question. Therefore, the likelihood function is not continuous in the parameters that define admissibility. As a consequence, classic gradient descent methods cannot be applied to find the best fitting parameters to define route admissibility. In fact, computing admissible routes is so computationally costly that an exhaustive search for the optimal route admissibility parameters is often impracticable.

Below, we will focus on the second step of the two step procedure outlined above. We will not provide details of how to compute admissible paths, as this is beyond the scope of this paper. Instead, we refer the interested reader to the paper by Fischer [1]. Throughout this appendix, we will assume that a suitable set of admissible routes has already been computed and focus on fitting the submodel that assigns probabilities to routes.

Recall that our survey effort is not uniformly distributed over all potential routes in general. Therefore, we have to consider where, when, and for how long we conducted surveys on the survey date. To measure the effect of survey timing, the temporal pattern model must be fitted before the route choice model. Similar to the survey timing, the compliance rate affects the route choice model, too, as we will see below. Therefore, the compliance model must be fitted before the route choice model as well.

If an agent appears in our data set, they must have been surveyed somewhere on the survey date. Let k_a^{obs} be the location where we observed agent a. With the compliance model, the temporal pattern model, the route choice model, and parameters θ_{Route} to be fitted, we can determine the probability $p_a^{\text{obs}}(\theta_{\text{Route}})$ to observe agent a in the survey shift conducted at location k_a^{obs} on the observation day. Furthermore, we can compute the probability $p_a^{\text{all}}(\theta_{\text{Route}})$ to observe agent a at *some* survey location operated on that day. This probability reflects the survey effort on the day of the observation and takes the lengths of the survey shifts into account. The quotient $p_a^{\text{obs}}/p_a^{\text{all}}$ is the probability that we observed the agent at location k_a^{obs} given that the agent was surveyed at some survey location operated that day. Consequently, the conditional likelihood function reads

$$\begin{aligned} L_{\text{Route}}(\theta_{\text{Route}}) &= \prod_a \mathbb{P}\left(\text{survey agent } a \text{ at location } k_a^{\text{obs}}|\theta_{\text{Route}},\ \text{agent } a \text{ surveyed on day } D_a\right) \\ &= \prod_a \frac{p_a^{\text{obs}}(\theta_{\text{Route}})}{p_a^{\text{all}}(\theta_{\text{Route}})}. \end{aligned} \tag{A2}$$

To compute p_a^{obs} and p_a^{all}, we have to recall the structure of our route choice model. If agent a is travelling from origin i to destination j, then the probability that agent a passes survey location k on their journey is

$$\rho_{ijk} \quad = \quad (1-\eta_{\text{c}}) \sum_{P\in\mathcal{P}_{ij}:k\in P} \frac{l_P^{-\lambda}}{\sum\limits_{\tilde{P}\in\mathcal{P}_{ij}} l_{\tilde{P}}^{-\lambda}} + \eta_{\text{c}}\eta_{\text{o}}. \tag{A3}$$

Here, $\mathcal{P}_{ij}$ is the set of admissible routes for the source-sink pair (i, j), l_P is the length of path P, and η_{c}, η_{o}, and λ are the parameters to be fitted. Recall that η_{c} is the probability that an agent chooses an inadmissible path, and η_{o} is the probability that agents driving on inadmissible paths are driving via any given location in the road network.

In subsection i, we will provide details of how equation (A3) is related to p_a^{obs} and p_a^{all}. Furthermore, in subsection ii, we will show how the likelihood function can be computed efficiently. Beforehand, however, we have to discuss issues that could result in non-informative models.

Avoiding dominant noise

Equation (A3) includes a noise term accounting for agents not driving on admissible routes. We assume that these agents choose the locations they pass randomly. If all traffic were random ($\eta_{\text{c}} = 1$) and all randomly driving agents were driving by all survey locations ($\eta_{\text{o}} = 1$), the probabilities to observe these agents would be maximized. However, with $\eta_{\text{c}} = \eta_{\text{o}} = 1$, our route choice model would be non-informative and misleading.

To avoid that maximizing the likelihood results in a non-informative model, we need to integrate additional information. We therefore assume that agents driving on an inadmissible route have not been surveyed more than once once. This makes models unfit in which agents drive on zig-zag routes via many survey locations.

We apply this assumption to our survey data only and not for potential model predictions. That is, the additional assumption does not affect our model but how we view our data set. Since survey locations are often far apart from each other, the additional assumption is typically true in practice. However, if we surveyed traffic at locations close to each other, the additional assumption could lead to wrong results. Nonetheless, tests with simulated observation data suggest that the error introduced by this additional assumption is small as long as only few agents travel on inadmissible routes.

Non-estimability of noise

Even if noise does not dominate the model, our noise model leads to identifiability issues. Our route choice model allows us to determine the correct ratio between traffic along admissible paths and the random traffic *observed* at survey locations. However, our data contain no information on how many agents are driving on inadmissible routes *without passing* any survey location. Therefore, the total share of agents driving on inadmissible routes remains unknown. Consequently, we can neither estimate the probability η_{c} that agents choose an inadmissible path nor the probability η_{o} that these agents are observed at a survey location.

This issue can be resolved by assuming that *most* of the traffic (e.g. 95%) follows admissible paths. We can obtain specific estimates of η_{c} and η_{o} only if we know the total daily number of driving agents for some donor-recipient pairs. This number, however, is usually unknown in large-scale systems.

We argue that it is reasonable to assume that most agents drive on admissible routes, unless models with a larger noise term fit the data significantly better. We fit our model constraining $\eta_{\text{c}} \leq 0.05$.

(i) Deriving the likelihood function of the route choice model

After providing an overview of the model fitting procedure and potential issues, we proceed to derive the concrete structure of the likelihood function to be maximized.

Consider an agent a travelling from i to j via survey location k on day D. Let ξ be the compliance rate, and let τ_{kD} be the probability that this agent passes the survey location while it is operated. The value of τ_{kD} depends on the length and the starting time of the survey shift conducted at location k on day D. As route choice and travel timing are assumed to be independent random choices, the probability to survey agent a at k on day D is given by $p_{ijkD}^{\text{obs}} = \rho_{ijk}\tau_{kD}\xi$. Recall that ρ_{ijk} is the probability that agent a drives via location k.

As discussed in the previous section, we make an additional assumption on agents travelling on inadmissible routes. Therefore, we cannot apply equation (A3) to determine ρ_{ijk} when we fit the model. To derive an expression for p^{obs}_{ijkD} based on the adjusted ρ_{ijk}, we start by considering agents travelling on inadmissible routes. As proposed above, we assume that such agents in our data set were not observed at more than one survey location. Hence, the probability that we observed such an agent $\tilde{a}$ on day D at location k (and not at any other operated survey location) is

$$\tilde{\eta}^{\text{o}}_{kD} \quad = \quad \xi\tau_{kD}\eta_{\text{o}} \prod_{\bar{k}\in L_D:\bar{k}\neq k} \left(1 - \xi\tau_{\bar{k}D}\eta_{\text{o}}\right), \tag{A4}$$

whereby L_D is the set of all survey locations operated on day D. Here,

$$\xi\tau_{kD}\eta_{\text{o}} \quad = \quad \mathbb{P}(\text{survey } \tilde{a} \text{ at location } k) \tag{A5}$$

$$1 - \xi\tau_{\bar{k}D}\eta_{\text{o}} \quad = \quad \mathbb{P}\left(\text{do not survey } \tilde{a} \text{ at location } \bar{k}\right) \tag{A6}$$

for any agent $\tilde{a}$ driving on an inadmissible route.

Now let us consider an agent a travelling from i to j on day D on an admissible *or* an inadmissible route. Recall that agents choose inadmissible routes with probability η_{c}. Consequently, the probability that agent a chooses an admissible route via location k is

$$(1 - \eta_{\text{c}}) \sum_{P\in\mathcal{P}_{ij}:k\in P} \frac{l_P^{-\lambda}}{\sum\limits_{\tilde{P}\in\mathcal{P}_{ij}} l_{\tilde{P}}^{-\lambda}}$$

and the probability that we surveyed a at k on day D is given by

$$p^{\text{obs}}_{ijkD} \quad = \quad \xi\tau_{kD}\left(1 - \eta_{\text{c}}\right) \sum_{P\in\mathcal{P}_{ij}:k\in P} \frac{l_P^{-\lambda}}{\sum\limits_{\tilde{P}\in\mathcal{P}_{ij}} l_{\tilde{P}}^{-\lambda}} + \eta_{\text{c}}\tilde{\eta}^{\text{o}}_{kD}. \tag{A7}$$

After computing p^{obs}_{ijkD}, we must determine the probability p^{all}_{ijD} to observe an agent travelling from i to j on day D at *some* location. Note that the distribution of travelling agents is independent of the day according to our model. The only reason why p^{all}_{ijD} depends on D is that surveys are conducted at different locations and times on different days.

We can split p^{all}_{ijD} into

$$p^{\text{all}}_{ijD} \quad = \quad \left(1 - \eta_{\text{c}}\right) p^{\text{adm}}_{ijD} + \eta_{\text{c}} p^{\text{inadm}}_{D}, \tag{A8}$$

whereby p^{adm}_{ijD} is the probability to observe an agent driving on an admissible route from i to j on day D, and p^{inadm}_{D} the respective probability for an agent driving along an inadmissible route. The value of p^{inadm}_{D} is independent of origin and destination of the considered agent.

We find p^{adm}_{ijD} by summing over all admissible paths $P \in \mathcal{P}_{ij}$ from i to j that go via a survey location $\tilde{k} \in L_D$ that is operated on day D. The probability to choose an admissible path $P \in \mathcal{P}_{ij}$ is given by

$$\mathbb{P}(\text{choose path } P \mid \text{driving on an admisible path}) \quad = \quad \frac{l_P^{-\lambda}}{\sum\limits_{\tilde{P}\in\mathcal{P}_{ij}} l_{\tilde{P}}^{-\lambda}}. \tag{A9}$$

The probability to observe an agent driving along this path at at least one operated survey location is

$$\mathbb{P}(\text{survey agent} \mid \text{driving on path } P \text{ on day } D) \quad = \quad 1 - \prod_{\bar{k}\in L_D:\bar{k}\in P} \left(1 - \xi\tau_{\bar{k}D}\right). \tag{A10}$$

Consequently,

$$p^{\text{adm}}_{ijD} \quad = \quad \sum_{P\in\mathcal{P}_{ij}:\tilde{k}\in P,\tilde{k}\in L_D} \frac{l_P^{-\lambda}}{\sum\limits_{\tilde{P}\in\mathcal{P}_{ij}} l_{\tilde{P}}^{-\lambda}} \left(1 - \prod_{\bar{k}\in L_D:\bar{k}\in P} \left(1 - \xi\tau_{\bar{k}D}\right)\right). \tag{A11}$$

After finding p^{adm}_{ijD}, we need to find an expression for p^{inadm}_{D}. This is the probability to observe an agent driving along an inadmissible path at exactly one of the survey locations operated on day D (compare with

equation (A4)):

$$p_D^{\text{inadm}} = \eta_o \sum_{\bar{k}\in L_D} \xi\tau_{\bar{k}D} \prod_{\hat{k}\in L_D:\hat{k}\neq\bar{k}} \left(1-\xi\tau_{\hat{k}D}\eta_o\right). \tag{A12}$$

Putting these pieces together we obtain the log-likelihood function

$$L(\theta) = \prod_{(ijkD)} \frac{p_{ijkD}^{\text{obs}}(\theta)}{p_{ijD}^{\text{all}}(\theta)} \tag{A13}$$

with p_{ijkD}^{obs} as defined in equation (A7) and p_{ijD}^{all} as defined in equation (A8) with the terms given in equations (A11) and (A12). Our goal is to find $\hat{\theta} = \left(\hat{\lambda}, \hat{\eta}_c, \hat{\eta}_o\right)$ that maximizes $L(\theta)$.

(ii) Computing the likelihood of the route choice model

During the likelihood maximization, we have to evaluate $L(\theta)$ and its derivatives many times. Computing $L(\theta)$ as derived above is expensive, because we have to compute nested products and sums. In this section, we show how the function can be split to speed up computations.

When we maximize the likelihood $L(\theta)$, we consider the log-likelihood $\ln L(\theta)$ to avoid numerical instabilities. However, we will work with the original likelihood function here for notational convenience.

To evaluate the likelihood function faster, we compute the following expressions first:

$$\begin{aligned}
\Xi_{DP} &= 1 - \prod_{\bar{k}\in L_D:\bar{k}\in P} \left(1-\xi\tau_{\bar{k}D}\right), & \Lambda_{ij}^{\text{norm}}(\lambda) &= \frac{1}{\sum_{\tilde{P}\in\mathcal{P}_{ij}} l_{\tilde{P}}^{-\lambda}},\\
\Phi_D(\eta_o) &= \prod_{\bar{k}\in L_D} \left(1-\xi\tau_{\bar{k}D}\eta_o\right), & \Lambda_{ijk}^{\text{spec}}(\lambda) &= \sum_{P\in\mathcal{P}_{ij}:k\in P} l_P^{-\lambda},\\
\Upsilon_D(\eta_o) &= \sum_{\bar{k}\in L_D} \frac{\xi\tau_{\bar{k}D}}{1-\eta_o\xi\tau_{\bar{k}D}}, & \Lambda_{ijD}^{\text{all}}(\lambda) &= \sum_{P\in\mathcal{P}_{ij}:\tilde{k}\in P,\tilde{k}\in L_D} l_P^{-\lambda}\Xi_{DP}.
\end{aligned} \tag{A14}$$

With these expressions, we can write

$$L(\lambda,\eta_c,\eta_o) = \prod_{(ijkD)} \frac{\xi\tau_{kD}\left((1-\eta_c)\Lambda_{ij}^{\text{norm}}(\lambda)\Lambda_{ijk}^{\text{spec}}(\lambda) + \frac{\eta_c\eta_o}{1-\eta_o\xi\tau_{kD}}\Phi_D(\eta_o)\right)}{(1-\eta_c)\Lambda_{ij}^{\text{norm}}(\lambda)\Lambda_{ijD}^{\text{all}}(\lambda) + \eta_c\eta_o\Upsilon_D(\eta_o)\Phi_D(\eta_o)}. \tag{A15}$$

Computing all required values of Ξ_{DP}, $\Phi_D(\eta_o)$, $\Upsilon_D(\eta_o)$, $\Lambda_{ij}^{\text{norm}}(\lambda)$, $\Lambda_{ijk}^{\text{spec}}(\lambda)$, and $\Lambda_{ijD}^{\text{all}}(\lambda)$ before the likelihood increases the computational efficiency.

Runtime analysis

To demonstrate how the function split speeds up the likelihood computation, we will now conduct a runtime analysis. To this end, we define count variables as follows:

n_{obs}: total number of surveyed agents
n_{pairs}: number of origin-destination pairs for which we have surveyed at least one agent
n_{days}: number of survey days
n_{loc}: number of survey locations
$n_{\text{pairs/day}}$: average daily number of origin-destination pairs for which we have surveyed at least one agent
$n_{\text{loc/day}}$: average number of survey locations operated on a survey day
$n_{\text{paths/pair}}$: average number of admissible routes between an origin and a destination

Let us start the runtime analysis by noting that Ξ_{DP} is independent of all parameters that we are optimizing. Therefore, we can compute Ξ_{DP} for all indices D and P before the optimization. For each survey day, we have to compute Ξ_{DP} for all admissible paths connecting origin-destination pairs for which we have observed an agent. Computing a single value of Ξ_{DP} requires $\mathcal{O}\left(n_{\text{loc/day}}\right)$ operations. Hence, we can compute Ξ_{DP} in $\mathcal{O}\left(n_{\text{days}}n_{\text{pairs/day}}n_{\text{paths/pair}}n_{\text{loc/day}}\right)$. Later, we can access the pre-computed values in effectively constant time.

To determine the values of Φ_D and Υ_D, we compute a product or sum over all survey locations operated on each day, respectively. This are $\mathcal{O}\left(n_{\text{days}} n_{\text{loc/day}}\right)$ operations.

The normalization constants $\Lambda_{ij}^{\text{norm}}$ for route choice probabilities must be computed for each origin-destination pair for which we have surveyed at least one agent. Computing a single $\Lambda_{ij}^{\text{norm}}$ value requires us to sum over all paths from the considered origin to the respective destination. Hence, we require $\mathcal{O}\left(n_{\text{pairs}} n_{\text{paths/pair}}\right)$ operations in total. The same applies to the computation of $\Lambda_{ijk}^{\text{spec}}$ with the exception that we have to consider a different set of paths for each observed combination of origin, destination, and survey location. Hence, computing all the $\Lambda_{ijk}^{\text{spec}}$ values requires $\mathcal{O}\left(n_{\text{pairs}} n_{\text{paths/pair}} n_{\text{loc}}\right)$ operations.

To compute the values of $\Lambda_{ijD}^{\text{all}}$, we conduct operations similar to those for $\Lambda_{ijk}^{\text{spec}}$. However, each survey day we may consider a different set of survey locations. Therefore, we need $\mathcal{O}\left(n_{\text{days}} n_{\text{pairs/day}} n_{\text{paths/pair}} n_{\text{loc/day}}\right)$ operations.

With all partial results determined, we can compute the likelihood in $\mathcal{O}(n_{\text{obs}})$ operations. We arrive at a final runtime of $\mathcal{O}\left(n_{\text{pairs}} n_{\text{paths/pair}} n_{\text{loc}} + n_{\text{days}} n_{\text{pairs/day}} n_{\text{paths/pair}} n_{\text{loc/day}} + n_{\text{obs}}\right)$, whereby the second summand is usually dominating. Note that $\mathcal{O}\left(n_{\text{days}} n_{\text{pairs/day}}\right)$ is bounded by $\mathcal{O}(n_{\text{obs}})$. Furthermore, $n_{\text{paths/pair}}$ and $n_{\text{loc/day}}$ are usually moderate numbers that are independent of the scale of the considered system and do not increase with the sample size. Thus, it is appropriate to classify the runtime of our algorithm as $\mathcal{O}(n_{\text{obs}})$, which is the sample size.

(d) Likelihood of the stochastic gravity model

In this section, we first state the optimization problem that must be solved to fit the gravity model to survey data. Then, we describe why this problem is computationally hard. In the second part of this section, we show how the structure of the likelihood function and the excess of observed zero counts can be exploited to compute the log-likelihood more efficiently.

(i) Deriving the likelihood function of the stochastic gravity model

We parameterize the negative binomial distribution for a random variable N by

$$\mathbb{P}\left(N = n\right) = f_{\text{NB}}\left(n \mid \mu, p\right) = \binom{n + r\left(\mu, p\right) - 1}{n} p^{r(\mu,p)} \left(1-p\right)^{n} \tag{A16}$$

with $r\left(\mu, p\right) := \frac{p}{1-p}\mu$. Here, $\mu = \mathbb{E}(N)$ is the mean of the random variable N, and $p = \frac{\mu}{\sigma^2}$ is the quotient of mean and variance of N. For convenience, we write $f_{\text{NB}}\left(n \mid r, p\right)$ below. The fitted value of μ can be obtained using the equation $\hat{\mu} = \frac{1-\hat{p}}{\hat{p}}\hat{r}$.

Let Ψ be the set of all considered origin-destination pairs, denoted by $(i, j) \in \Psi$. We assume that on each day, the number of travelling agents for each origin-destination pair $(i, j) \in \Psi$ is negative binomially distributed with parameters r_{ij} and p. The parameter r_{ij} depends on the origin-destination pair (i, j), because we estimate the mean number of travelling agents with a gravity model that depends on the properties of origins and destinations. The parameter p, however, is assumed to be similar for all origin-destination pairs.

Let us index survey shifts with $s \in S$, whereby S is the set of all survey shifts. Each survey shift $s \in S$ is conducted at a location k_s and in a time interval $t_s = \left[t_s^{\text{start}}, t_s^{\text{end}}\right]$. Let ρ_{ijk_s} be the probability that an agent travelling from origin i to destination j chooses a path via location k_s. Furthermore, let τ_s be the probability that an agent travelling via k_s passes the survey location k_s during the time interval t_s the survey was conducted, and let ξ be the compliance rate. Lastly, let n_{ijs} be the number of agents travelling from i to j who were surveyed in shift s.

In our hierarchical stochastic model, the number N_{ijs} of agents travelling between the origin-destination pair $(i, j) \in \Psi$ and observed in shift $s \in S$ is distributed as

$$N_{ijs} \sim \textit{Binomial}\left(\textit{Binomial}\left(\textit{Binomial}\left(\textit{NegativeBinomial}\left(r_{ij}, q\right), \rho_{ijk_s}\right), \tau_s\right), \xi\right). \tag{A17}$$

Define $\tilde{p}_{ijs} := \frac{p}{p + \rho_{ijk_s}\tau_s\xi(1-p)}$. It can be shown [2] that

$$\mathbb{P}\left(N_{ijs} = n_{ijs}\right) = f_{\text{NB}}\left(n_{ijs} \mid r_{ij}, \tilde{p}_{ijs}\right). \tag{A18}$$

We desire to maximize the likelihood

$$L(\theta) \quad = \prod_{(i,j,s)\in\Psi\times S} f_{\mathrm{NB}}\left(n_{ijs}\,|\,r_{ij}(\theta),\tilde{p}_{ijs}(\theta)\right), \tag{A19}$$

whereby θ is a vector of parameters.

(ii) Computing the likelihood of the stochastic gravity model

To compute the likelihood given in equation (A19), we have to consider $|\Psi|\,|S|$ combinations of origin-destination pairs and survey shifts. This is a very large number in general. For example, in the application section of this paper, we considered about $|\Psi|\approx 3.7\cdot 10^5$ origin-destination pairs and $|S|\approx 1600$ survey shifts. Though computing $f_{\mathrm{NB}}\left(n_{ijs}\,|\,r_{ij}(\theta),\tilde{p}_{ijs}(\theta)\right)$ for all combinations of i, j, and s might be feasible, it takes too much time for a multidimensional optimization. To maximize the likelihood with reasonable effort, we would need to evaluate L within fractions of a second. Below, we present a way to speed up the likelihood computation.

Given agent counts n_{ijs}, the log-likelihood function reads

$$\ell(\theta) \quad = \sum_{(i,j,s)\in\Psi\times S} \ln f_{\mathrm{NB}}\left(n_{ijs}\,|\,r_{ij}(\theta),\tilde{p}_{ijs}(\theta)\right). \tag{A20}$$

The probability mass function $f_{\mathrm{NB}}\left(n_{ijs}\,|\,r_{ij}(\theta),\tilde{p}_{ijs}(\theta)\right)$ is particularly simple to compute in two cases: (1) if there is no admissible path from i to j via the survey location k_s, and (2) if $n_{ijs}=0$. Typically, most of the observations fall in one of these categories. We exploit that in the following way:

(i) We assume that *all* observations satisfy the criterion (1) or (2), respectively, and compute the log-likelihood under this assumption.
(ii) We consider all the data for which the assumption above was incorrect and compute the actual likelihood for these count values.
(iii) We determine the portion of the likelihood that we computed in step 1 under a wrong assumption. Then we replace this part of the likelihood with the correct likelihood computed in step 2.

Before we provide further details, we introduce some helpful notation.

(ii.1) Some notes on notation

Let $\Omega=\Psi\times S$ be the set of the indices of all observations. For convenience, we label the following logical statements as given below:

- Statement "0": $n_{ijs}=0$
- Statement "e": $\exists P\in\mathcal{P}_{ij}: k_s\in P$.

Recall that $\mathcal{P}_{ij}$ is the set of all admissible paths from i to j.

To denote that all elements in an index set satisfy a certain logical statement, we attach a corresponding subscript to the set. For example, $\Omega_0\subseteq\Omega$ is the subset of Ω for which all elements satisfy statement "0":

$$\Omega_0 \quad = \quad \left\{(i,j,s)\in\Omega\,|\,n_{ijs}=0\right\}. \tag{A21}$$

That is, Ω_0 contains the indices of zero counts. Similarly,

$$\Omega_e \quad = \quad \left\{(i,j,s)\in\Omega\,|\,\exists P\in\mathcal{P}_{ij}: k_s\in P\right\} \tag{A22}$$

contains the indices of all counts of agents surveyed at one of their admissible routes. That is, observations in Ω_e do not have to be considered traffic noise. Instead, these agents were observed where we expected them. Hence, we labelled the corresponding logical statement "e" for "expected".

We can use the same subscript notation to denote intersections, unions, and complements of sets. Recall that for any logical statements A and B, "$\neg A$" means "*not A*", $A\vee B$ means "*A or B*", and "$A\wedge B$" means "*A and B*". Thus, for example, $\Omega_{\neg 0}=\Omega\backslash\Omega_0$, $\Omega_{\neg e}=\Omega\backslash\Omega_e$, $\Omega_{0\wedge e}=\Omega_0\cap\Omega_e$, and $\Omega_{0\vee e}=\Omega_0\cup\Omega_e$.

Below, we are going to compute the log-likelihood ℓ under specific assumptions about our data. To show which data we are considering, respectively, we use a *subscript*. For example,

$$\ell_{\Omega_e}(\theta) = \sum_{(i,j,s)\in\Omega_e} \ln f_{\mathrm{NB}}\left(n_{ijs} \,|\, r_{ij}(\theta), \tilde{p}_{ijs}(\theta)\right) \tag{A23}$$

denotes the log-likelihood of data with indices in Ω_e.

Furthermore, we use a *superscript* to denote under which assumption we compute the log-likelihood. For example, if we compute the log-likelihood of all data under the assumption that we only observed zeros, we write

$$\ell^0_{\Omega}(\theta) = \sum_{(i,j,s)\in\Omega} \ln f_{\mathrm{NB}}\left(0 \,|\, r_{ij}(\theta), \tilde{p}_{ijs}(\theta)\right). \tag{A24}$$

Note that we iterated over *all* data here. That is, we included non-zero counts and assumed (falsely) that they *were* zero.

(ii.2) Splitting the log-likelihood

After introducing the required notation, we now proceed explaining how the log-likelihood can be computed more efficiently. Observe that for any logical statement A,

$$\ell_{\Omega}(\theta) = \ell^A_{\Omega}(\theta) - \ell^A_{\Omega_{\neg A}}(\theta) + \ell_{\Omega_{\neg A}}(\theta). \tag{A25}$$

That is, if we compute the log-likelihood under some assumption A, subtract the portion of this quantity for which the assumption was wrong, and add the correct log-likelihood value for these data instead, then we obtain the correct log-likelihood value.

Applying this observation and basic set operations, we obtain

$$\ell_{\Omega}(\theta) = \ell^{0\wedge\neg e}_{\Omega}(\theta) - \ell^{0\wedge\neg e}_{\Omega_{\neg 0\vee e}}(\theta) + \ell_{\Omega_{\neg 0\vee e}}(\theta) \tag{A26}$$

$$\ell^{0\wedge\neg e}_{\Omega_{\neg 0\vee e}}(\theta) = \ell^{0\wedge\neg e}_{\Omega_e}(\theta) + \ell^{0\wedge\neg e}_{\Omega_{\neg 0\wedge\neg e}}(\theta) \tag{A27}$$

$$\ell_{\Omega_{\neg 0\vee e}}(\theta) = \ell_{\Omega_e}(\theta) + \ell_{\Omega_{\neg 0\wedge\neg e}}(\theta) \tag{A28}$$

$$\ell_{\Omega_e}(\theta) = \ell^0_{\Omega_e}(\theta) - \ell^0_{\Omega_{\neg 0\wedge e}}(\theta) + \ell_{\Omega_{\neg 0\wedge e}}(\theta) \tag{A29}$$

Inserting these equations into each other yields

$$\ell_{\Omega}(\theta) = \ell^{0\wedge\neg e}_{\Omega}(\theta) - \ell^{0\wedge\neg e}_{\Omega_e}(\theta) - \ell^{0\wedge\neg e}_{\Omega_{\neg 0\wedge\neg e}}(\theta) + \ell^0_{\Omega_e}(\theta) - \ell^0_{\Omega_{\neg 0\wedge e}}(\theta) + \ell_{\Omega_{\neg 0\wedge e}}(\theta) + \ell_{\Omega_{\neg 0\wedge\neg e}}(\theta). \tag{A30}$$

The likelihood components on the right hand side of equation (A30) are easy to compute, because they have either a simple functional form or consider only a small fraction of our data. Most of our observations are in Ω_0 and/or Ω_e.

(ii.3) Computing the log-likelihood

To compute the log-likelihood we determine all the individual components of equation (A30) and insert them into the equation. Below, we describe how to compute each of the components efficiently.

$\ell^{0\wedge\neg e}_{\Omega}(\theta)$: If none of our survey locations were on any admissible route (statement "$\neg e$"), then the probability that an agent travels from i to j via a survey location k_s is

$$\rho_{ijk_s} = \eta_{\mathrm{C}}\eta_{\mathrm{O}}, \tag{A31}$$

which is independent of origin, destination, and survey location. It follows that $\tilde{p}_{ijs} = \frac{p}{p+\rho_{ijk}\tau_s(1-p)} = \frac{p}{p+\eta_{\mathrm{C}}\eta_{\mathrm{O}}\tau_s(1-p)} = \tilde{p}_s$ is independent of the considered source-sink pair (i, j). If we furthermore assume that no agent has been observed (statement "0"), then the likelihood function is given by

$$L^{0\wedge\neg e}_{\Omega}(\theta) = \prod_{s\in S}\prod_{(i,j)\in\Psi} \binom{0 + r_{ij}(\theta) - 1}{0} \left(\tilde{p}_s(\theta)\right)^{r_{ij}} \left(1 - \tilde{p}_s(\theta)\right)^0, \tag{A32}$$

and the log-likelihood is

$$\ell_{\Omega}^{0\wedge\neg e}(\theta) = \left(\sum_{s\in S}\ln\left(\tilde{p}_s(\theta)\right)\right)\left(\sum_{(i,j)\in\Psi} r_{ij}(\theta)\right). \tag{A33}$$

We can compute this value in $\mathcal{O}(|S|+|\Psi|)$ steps.

$\ell_{\Omega_e}^{0\wedge\neg e}(\theta)$: Let $\Psi_k = \left\{(i,j)\in\Psi \mid \exists P\in\mathcal{P}_{ij} : k\in P\right\}$ be the set of origin-destination pairs for which at least one admissible path $P\in\mathcal{P}_{ij}$ passes survey location k. Let furthermore $\tilde{r}_k = \sum_{ij\in\Psi_k} r_{ij}$ be the sum of the r-parameters for these pairs. Then it is easy to compute

$$\ell_{\Omega_e}^{0\wedge\neg e}(\theta) = \sum_{s\in S}\tilde{r}_{k_s}\ln\left(\tilde{p}_s(\theta)\right). \tag{A34}$$

Computing the values of $\tilde{r}_k$ for all used survey sites before evaluating equation (A34) saves the efforts of computing the same quantity multiple times. The worst-case runtime for computing the values of r_k is $\mathcal{O}(|L|\,|\Psi|)$, whereby L is the set of all survey locations. Computing $\ell_{\Omega_e}^{0\wedge\neg e}(\theta)$ runs in $\mathcal{O}(|S|+|L|\,|\Psi|)$.

$\ell_{\Omega_{\neg 0\wedge\neg e}}^{0\wedge\neg e}(\theta)$: The set $\Omega_{\neg 0\wedge\neg e}$ contains the indices of those observations where the agents were certainly driving along inadmissible routes. As most agents drive along admissible routes, the set $\Omega_{\neg 0\wedge\neg e}$ is small. Hence, we do not need any optimizations to compute the value of $\ell_{\Omega_{\neg 0\wedge\neg e}}^{0\wedge\neg e}(\theta)$:

$$\ell_{\Omega_{\neg 0\wedge\neg e}}^{0\wedge\neg e}(\theta) = \sum_{(i,j,s)\in\Omega_{\neg 0\wedge\neg e}} r_{ij}\ln\left(\tilde{p}_{ijs}(\theta)\right). \tag{A35}$$

This runs in $\mathcal{O}(|\Omega_{\neg 0\wedge\neg e}|)$.

$\ell_{\Omega_e}^{0}(\theta)$: Computing the value of $\ell_{\Omega_e}^{0}(\theta)$ may be the most challenging part of the likelihood computation, as the set Ω_e is large and the likelihood function is not simple. Therefore, we apply Taylor approximations in $\nu_s := \xi\tau_s$ to split the nested sums into separate sums that can be computed more efficiently. The approximation point of the Taylor expansion will be the mean $\bar{\nu} := \frac{\xi}{|S|}\sum_{s\in S}\tau_s$. We get

$$\begin{aligned}
\ell_{\Omega_e}^{0}(\theta) &= \sum_{s\in S}\sum_{(i,j)\in\Psi_{k_s}} r_{ij}\ln\left(\tilde{p}_{ijs}\right)\\
&= \sum_{s\in S}\sum_{(i,j)\in\Psi_{k_s}} r_{ij}\ln\left(\frac{p}{p+(1-p)\rho_{ijk_s}\nu_s}\right)\\
\overset{\text{Taylor expansion}}&{\approx} \sum_{s\in S}\sum_{(i,j)\in\Psi_{k_s}} r_{ij}\left(\ln\left(\frac{p}{p+(1-p)\rho_{ijk_s}\bar{\nu}}\right)+\sum_{m=1}^{M}\frac{1}{m}\left(\frac{-(1-p)\rho_{ijk_s}(\nu_s-\bar{\nu})}{p+(1-p)\rho_{ijk_s}\bar{\nu}}\right)^m\right)\\
&= \sum_{s\in S}\sum_{(i,j)\in\Psi_{k_s}} r_{ij}\ln\left(\frac{p}{p+(1-p)\rho_{ijk_s}\bar{\nu}}\right)\\
&\quad +\sum_{s\in S}\sum_{m=1}^{M}\frac{1}{m}(\nu_s-\bar{\nu})^m\sum_{(i,j)\in\Psi_{k_s}} r_{ij}\left(\frac{-(1-p)\rho_{ijk_s}}{p+(1-p)\rho_{ijk_s}\bar{\nu}}\right)^k\\
&= \sum_{s\in S}R_{k_s}+\sum_{s\in S}\sum_{m=1}^{M}\frac{1}{m}(\nu_s-\bar{\nu})^m\tilde{R}_{k_s m}
\end{aligned} \tag{A36}$$

with

$$R_k = \sum_{(i,j)\in\Psi_k} r_{ij}\ln\left(\frac{p}{p+(1-p)\rho_{ijk}\bar{\nu}}\right), \tag{A37}$$

$$\tilde{R}_{km} = \sum_{(i,j)\in\Psi_k} r_{ij}\left(\frac{-(1-p)\rho_{ijk}}{p+(1-p)\rho_{ijk}\bar{\nu}}\right)^m. \tag{A38}$$

Note that p and r_{ij}, and therefore also $\tilde{p}_{ij}$, R_k, and $\tilde{R}_{km}$ depend on θ. The parameter M determines the precision of the Taylor approximation.

We can estimate the error introduced by the Taylor approximation by considering the term $\frac{-(1-p)\rho_{ijk_s}(\nu_s-\bar{\nu})}{p+(1-p)\rho_{ijk_s}\bar{\nu}}$. Recall that ν_s, ρ_{ijk_s}, and p can be interpreted as probabilities and are therefore bounded between 0 and 1. Consequently, choosing $\bar{\nu}=\frac{1}{2}$ would imply $|\nu_s-\bar{\nu}|\leq\frac{1}{2}$. In this case,

$$\left|\frac{-(1-p)\,\rho_{ijk_s}\,(\nu_s-\bar{\nu})}{p+(1-p)\,\rho_{ijk_s}\bar{\nu}}\right| \quad\leq\quad \frac{(1-p)\,\rho_{ijk_s}\bar{\nu}}{p+(1-p)\,\rho_{ijk_s}\bar{\nu}}, \tag{A39}$$

which is a function decreasing in p and increasing in ρ_{ijk_s}. As $p=\frac{\mu}{\sigma^2}>0$, we know that the full term is less than 1, which in turn guarantees that the series converges. Moreover, it is reasonable to assume that the overdispersion is not extreme and $p=\frac{\mu}{\sigma^2}\geq\frac{1}{10}$. This would imply that

$$\left|\frac{-(1-p)\,\rho_{ijk_s}\,(\nu_s-\bar{\nu})}{p+(1-p)\,\rho_{ijk_s}\bar{\nu}}\right| \quad\leq\quad \frac{9}{11}, \tag{A40}$$

and the error would be bounded by a quantity proportional to $\frac{1}{M+1}\left(\frac{9}{11}\right)^{M+1}$. In practice, the error can be checked by investigating the change in the computed log-likelihood as M is increased. In our application, the error was small for $M=3$.

To see how the Taylor expansion simplifies the computation, note that both R_k and $\tilde{R}_{km}$ do not have to be computed for each survey shift $s\in S$ but rather for each used survey location $k\in L$. Therefore, computing these values runs in $\mathcal{O}(M\,|L|\,|\Psi|)$. Evaluating the right hand side of equation (A36) runs in $\mathcal{O}(M\,|S|)$. Thus, the Taylor expansion allows us to compute $\ell^0_{\Omega_e}(\theta)$ in $\mathcal{O}(M\,|L|\,|\Psi|+M\,|S|)$ instead of $\mathcal{O}(|\Psi|\,|S|)$.

$\ell^0_{\Omega_{\neg 0\wedge e}}(\theta)$: As the number of non-zero counts is moderate, so is $|\Omega_{\neg 0\wedge e}|$. Therefore, we could compute $\ell^0_{\Omega_{\neg 0\wedge e}}(\theta)$ without further optimizations. However, we compute $\ell^0_{\Omega_{\neg 0\wedge e}}(\theta)$ to reduce $\ell^0_{\Omega_e}(\theta)$ by the amount corresponding to the data for which statement "0" was incorrect. Therefore, we apply the same Taylor approximation as above. That is,

$$\begin{aligned}\ell^0_{\Omega_{\neg 0\wedge e}}(\theta) &= \sum_{(i,j,s)\in\Omega_{\neg 0\wedge e}} r_{ij}\ln\left(1-\tilde{q}_k(\theta)\right)\\ &\approx \sum_{(i,j,s)\in\Omega_{\neg 0\wedge e}} r_{ij}\left(\ln\left(\frac{p}{p+(1-p)\,\rho_{ijk_s}\bar{\nu}}\right)\right.\\ &\qquad\left.+\sum_{m=1}^{M}\frac{1}{m}\left(\frac{-(1-p)\,\rho_{ijk_s}\,(\nu_s-\bar{\nu})}{p+(1-p)\,\rho_{ijk_s}\bar{\nu}}\right)^m\right).\end{aligned} \tag{A41}$$

This computation runs in $\mathcal{O}(M\,|\Omega_{\neg 0\wedge e}|)$.

$\ell_{\Omega_{\neg 0\wedge e}}(\theta)$: The number of non-zero observations is small. Therefore, we can compute $\ell_{\Omega_{\neg 0\wedge e}}(\theta)$ directly:

$$\ell_{\Omega_{\neg 0\wedge e}}(\theta) \quad=\quad \sum_{(i,j,s)\in\Omega_{\neg 0\wedge e}} \ln f_{\mathrm{NB}}\left(n_{ijs}\,|\,r_{ij}(\theta),\tilde{p}_{ijs}(\theta)\right), \tag{A42}$$

which runs in $\mathcal{O}(|\Omega_{\neg 0\wedge e}|)$.

$\ell_{\Omega_{\neg 0\wedge\neg e}}(\theta)$: We have already noted that the set $\Omega_{\neg 0\wedge\neg e}$ of traffic noise observations is small. Therefore, we compute $\ell_{\Omega_{\neg 0\wedge\neg e}}(\theta)$ directly:

$$\ell_{\Omega_{\neg 0\wedge\neg e}}(\theta) \quad:=\quad \sum_{(i,j,s)\in\Omega_{\neg 0\wedge\neg e}} \ln f_{\mathrm{NB}}\left(n_{ijs}\,|\,r_{ij}(\theta),\tilde{p}_{ijs}(\theta)\right). \tag{A43}$$

This runs in $\mathcal{O}(|\Omega_{\neg 0\wedge\neg e}|)$.

In conclusion, we can compute the likelihood in $\mathcal{O}(M\,|S|+M\,|L|\,|\Psi|+M\,|\Omega_{\neg 0}|)$. The set $\Omega_{\neg 0}$ contains all those observations that are non-zero and has a size proportional to $|S|$ in general.

There are more (minor) optimizations that can be applied to compute intermediate terms independent of θ before the optimization process. We do not list these details here.

(e) Maximizing the likelihood

We apply different optimization techniques consecutively to maximize the likelihood. All algorithms we used were implemented in the Scipy package "optimize" version 1.0 [3]. We started with the "differential evolution" algorithm by Storn and Price [4], a meta-heuristic global optimization algorithm that does

not require an initial guess. We chose the region of admissible parameters liberally. With the differential evolution result as initial guess, we applied the L-BFGS-G algorithm [5], which proved to be robust and efficient even if the result from the genetic algorithm was far from the optimum. In a next step, we applied sequential least squares programming [6] due to its high efficiency and finally a trust-region Newton-Raphson method [7], which is guaranteed to converge very fast if the initial guess is close to the optimum.

Whenever necessary, we determined derivatives of the likelihood function using algorithmic differentiation in reverse mode, which is much more efficient and precise than numerical differentiation. We used the python package "autograd" for this task.

Though some of the optimization algorithms we applied can deal with constraints on parameters, we enforced constraints with parameter transformations. Let c be a parameter as it appears in the model, and $\tilde{c}$ the same parameter as used in computations.

- Parameters constrained to be positive were expressed as

$$c \quad = \quad \begin{cases} \exp \tilde{c} & \text{if } \exp \tilde{c} < 0 \\ \tilde{c} + 1 & \text{else.} \end{cases} \tag{A44}$$

- Parameters constrained to the interval $(0, 1]$ were expressed as $c = \frac{1}{\pi} \arctan(\tilde{c}) + \frac{1}{2}$.

This allowed us to avoid numerical instabilities arising when the results are close to the boundaries.

E. Model selection and confidence intervals based on the composite likelihood

(a) Model selection

We used an information criterion to determine which of our gravity models fits the data best without overfitting. The most widely used criteria for model selection [8] are the information criterion by Akaike (AIC, 9) and the Bayesian information criterion (BIC, 10). Both AIC and BIC are based on the log-likelihood of the compared models.

When working with composite likelihood, as we did to determine the best structure for the gravity model, AIC and BIC lose their validity [11]. However, the corrected information criterion that Varin and Vidoni [11] derived for composite likelihood models is hard to compute. Furthermore, only a small portion of our data violate the independence assumption. Therefore, we proceeded using the classical model selection criteria.

(b) Confidence intervals

For practical reasons, we computed the confidence intervals for our parameters (see Tables **??** and **??** below) under simplifying assumptions. The first simplification is that we determined the confidence intervals for each submodel individually. This approach measures the credibility of the fitted parameters under the assumption that the previously fitted submodels are known. However, if all submodels were fitted simultaneously, changes to the parameters of one model would also affect the parameter estimates for the other model. Consequently, the confidence intervals would increase. The second simplification is that we computed the confidence intervals based on the composite likelihood. Though this does not bias our parameter estimate, more sophisticated methods would be necessary to determine the confidence intervals accurately [12].

Though these limitations decrease the rigorous meaning of the confidence intervals we computed, the presented confidence intervals still provide valuable insights into the levels of credibility of our estimates, since only small portions of our data are dependent on each other.

F. Model validation

In this appendix, we present model validation results and the methods that we applied to obtain these results. Specifically, we confirm that the distribution choices for our temporal pattern model (von Mises distribution) and the count data (negative binomial distribution) are appropriate. Furthermore, we check our

model for an overall bias and assess the precision of the model's predictions. We start with a description of our methods, continue with the results, and conclude the Appendix with a brief discussion of both validation results and methods.

(a) Methods

Before we start describing our methods in detail, we make a general note on model validation. In general, it is hard to apply classical hypothesis testing for model validation, as the distribution of the data under the null hypothesis “model is incorrect” is unknown. We therefore validate our model by ascertaining that it cannot be rejected on a high confidence level. That is, our null hypothesis is “model is correct”, and high p-values indicate that the test statistic results computed with the data we observed are likely to occur if the model is correct. Though this method can provide some insights into whether the model is appropriate, the approach does not yield a rigorous measure for the model validity. Therefore, we also perform validation steps based on graphical comparison.

(i) Homogenized samples

Some of the tests we are about to apply require samples from count data distributions. That is, we need a set of independent and identically distributed (i.i.d.) observations. Both the survey location and the survey time affect the distribution of count data of observed agents. Therefore, we will get i.i.d. observations only if we consider count data collected at the same survey location and during the same time interval.

We generated such samples by considering count data collected in a time interval that overlapped with many of our observation shifts. We proceeded as follows:

(i) We considered all survey shifts that started at 11AM or earlier and ended at 4PM or later. We neglected all other survey shifts.
(ii) For each of the above survey shifts, we counted the agents surveyed between 11AM and 4PM.
(iii) For each survey location, we noted how many survey shifts were considered in step 2. To ensure we had enough data for a meaningful statistical analysis, we neglected samples with sizes below 20.

(ii) Shape of the temporal traffic pattern

In this section, we describe a test to check whether our temporal traffic model has an appropriate shape. We used the von Mises distribution to model the temporal variations of agent traffic. This distribution has a specific unimodal shape. This shape may differ significantly from the observed traffic profile, which may have multiple peaks.

To ensure that the von Mises distribution is appropriate to model the daily traffic pattern, we compared it to fitted step function distributions, which do not have a predefined shape. Let $I_{\text{tot}} = [t_0, t_n]$ denote the portion of the day that was covered by at least one survey shift. A step function distribution splits I_{tot} in n equally sized disjoint intervals $I_1, \ldots, I_n \subseteq I_{\text{tot}}$ with $I_i = [t_{i-1}, t_i)$. The probability density function is given by

$$f_{\text{step}}\left(t \middle| p_1, \ldots p_n\right) = \begin{cases} p_1 & \text{if } t \in I_1 \\ \vdots & \vdots \\ p_n & \text{if } t \in I_n. \end{cases} \tag{A1}$$

We fitted the parameters p_i with a maximum likelihood approach and repeated this procedure for distributions with different interval numbers n. Then, we compared the resulting AIC values and probability density functions with the best-fit von Mises distribution. Both the AIC value and graphical comparison yield insights into whether the von Mises distribution is appropriate. Note that a non-parametric estimate of the temporal traffic distribution is not easily possible because the survey effort was not uniform over the day.

(iii) Distribution of count data

In this section, our goal is to check whether the negative binomial distribution is appropriate to model the distribution of our count data. To that end, we use the homogenized count data described in section i. We have count data $x_i = \{x_{i1}, \ldots, x_{in_i}\}$ for different origin destination pairs, obtained at different locations.

Here, i enumerates all combinations of origins, destinations and sampling locations for which we have sufficient data. The numbers $n_i \in \mathbb{N}$ denote the respective sample sizes. Below, we write X_i for the random variable that x_i has been drawn from.

Since we expect that both the sampling location as well as origin and destination affect the count distribution, we need to check that all data come from negative binomial distributions, i.e. $X_i \sim NB(\mu_i, p_i)$, without assuming that all data come from the *same* distribution. That is, μ_i and p_i may differ dependent on i. In this section, we describe a method to test this hypothesis.

Famoye [13] compared the power of different empirical distribution function tests to test whether observations come from a generalized negative binomial distribution. In their simulations, the discrete Anderson-Darling test performed best. The Anderson-Darling test compares the cumulative mass function (cmf) of a null distribution with its empirical counterpart generated from the considered sample. Thereby, the Anderson-Darling test puts higher weight on the tails of the distribution than other comparable tests, like the Kolmogorov-Smirnov test. If the empirical and the hypothesized cmf differ significantly, the null hypothesis is rejected.

The distribution of the Anderson-Darling statistic is known for fully specified continuous null distributions. We, however, consider a discrete distribution and do not have prior knowledge of the parameters. Instead, we are only interested in whether the observed data come from *some* negative binomial distribution. To generate the cmf of the null distribution, which is needed for comparison with the empirical cmf, we would have to estimate the distribution's parameters first. This, in turn, affects the distribution of the test statistic.

We are not aware of any result providing a closed-form expression for the distribution of the Anderson-Darling statistic applied to negative binomial random variables. Therefore, we determine the p-values for our samples by adjusting the the parametric bootstrap procedure used by Famoye [13]. Parametric bootstrap methods approximate the distribution of a test statistic by repeated application of the statistic to samples randomly generated from the null distribution. Therefore, parametric bootstrap methods are not exact but easy to implement.

The p-value of a test statistic T applied to a sample x_i is the probability to observe $T(x_i)$ if the null hypothesis is true. Consequently, a high p-value indicates that the null distribution may be appropriate to model the data. Thus it seems reasonable to assume that if the p-values for all individual samples x_i, $i \in \{1, \ldots, N\}$, are large, the null distribution can be assumed to be a good model for all our count data. This is the main idea of our approach.

Note that since each computed p-value depends on the randomly drawn sample x_i, the p-values itself are random variables as well. To test our count distribution hypothesis on all N samples, we may check whether the N computed p-values come from the distribution of p-values that we would expect under the null hypothesis. By this means, we could summarize all individual tests in one joint test.

For such a joint test, we need to know the distribution of p-values under the null hypothesis. Since, by construction of the p-value, 80% of the samples randomly drawn from the null distribution lead to a p-value less than or equal to 0.8, 60% of the samples lead to a p-value less than or equal to 0.6, and so on, it is intuitive to assume that the p-values follow a uniform distribution on the interval $(0, 1]$. This is in fact true for continuous null distributions. For samples from discrete distributions, however, things are more complicated.

Discrete random variables attain their values with positive probabilities. Hence, the same applies to samples drawn from this distribution and thus for computed p-values. Suppose, for example, that we have drawn a sample x_i from the null distribution and computed the p-value $\phi(x_i)$, say $\phi(x_i) = 1$. Then the probability to obtain a p-value of 1 is at least $\mathbb{P}(x_i)$, which could be arbitrarily high. In fact, since samples taken from a single distribution are permutation-invariant, $\mathbb{P}(\phi\,(X_i) = 1)$ can attain relatively large numbers in practice. Therefore, the distribution of $\phi\,(X_i)$ may not even be close to a uniform distribution, and we have to determine the distribution of $\phi\,(X_i)$ under the null hypothesis before we can test our joint hypothesis.

We present our overall approach by breaking it down into parts. First, we describe the parametric bootstrap algorithm we use to compute p-values for a single sample x_i. Then, we show how we estimate the joint distribution of the p-values for all samples $x_1, \ldots, x_N$. Third, we describe how a second parametric bootstrap procedure can be applied to compute the p-value for our joint hypothesis. In a fourth step, we study the distribution of our count data samples under the null hypothesis and provide computationally efficient parameter estimators. Fifth, we describe how random numbers can be drawn from the null distribution. We conclude this section by showing how partial results can be reused to speed up computations and discussing how the accuracy of the resulting p-value can be determined.

(iii.1) Computing p-values with the Anderson-Darling test for a null distribution with unknown parameters

In this subsection, we describe the parametric bootstrap procedure based on Famoye [13] that we apply to determine the p-values for the Anderson-Darling tests for individual samples. Let $T(x_i, \theta)$ be the function that maps a sample x_i to the Anderson-Darling statistic based on the null distribution with parameters θ. Furthermore, let $\Theta(x_i)$ be an estimate of the parameters θ of the null distribution based on sample x_i. Let x_0 be the sample that we want to study, $n := |x_0|$, and $M_1 \in \mathbb{N}_+$ be a positive integer. Throughout this Appendix, $|A|$ denotes the number of entries in a vector or set A.

The parametric bootstrap method works as follows:

(i) Use the sample x_0 to find an estimate $\hat{\theta}_0 := \Theta(x_0)$ of the parameters of the null distribution.
(ii) Compute the test statistic $t_0 := T(x_0, \hat{\theta}_0)$ under the null distribution with the fitted parameters.
(iii) Generate M_1 samples $\tilde{x}_i$, $i := 1, \ldots, M_1$, of size n from the null distribution with parameters $\hat{\theta}_0$.
(iv) For each generated sample $\tilde{x}_i$:
   (a) Find an estimate of the parameters $\hat{\theta}_i := \Theta(\tilde{x}_i)$ based on sample $\tilde{x}_i$.
   (b) Compute $t_i := T(\tilde{x}_i, \hat{\theta}_i)$.
(v) The approximate p-value is given by the fraction of samples that had an at least equally large test statistic: $\phi(x_0) := \frac{1}{M_1}\left|i : t_i \geq t_0\right|$.

(iii.2) Determining the null distribution of p-values

To test which distribution of p-values we would expect under the null distribution, we apply a Monte Carlo simulation. That is, we draw many samples from the null distribution and determine the respective p-values. Then, we determine the empirical distribution function of these samples.

Recall that the null-distribution of p-values may be different for each sample x_i, because we do not require that all samples come from the same distribution. Therefore, the true distribution of p-values is a multivariate distribution. However, to compute a statistic from the samples, we have to reduce the dimension somehow. We therefore consider the random variable Φ resulting from the following random process:

(i) Choose $i \in \{1, \ldots, N\}$ randomly from a uniform distribution.
(ii) Set $\Phi = \phi(X_i)$.

That is, we suppose that Φ assumes p-values from each dimension with the same probability.

To ease the explanation of our method, let us now assume that the parameters θ_i of the null distribution for sample i are known, i.e., that the null distribution is fully specified. We will extend the method to not fully specified null distributions in the next section.

Let $x_1, \ldots, x_N$ be our count data samples, and let $n_i := |x_i|$ and $M_2 \in \mathbb{N}_+$ be a positive integer. To determine the distribution of Φ under the null hypothesis, we proceed as follows:

(i) For $i \in \{1, \ldots, N\}$:
   (a) Given the parameters θ_i of the null distribution for sample i, draw M_2 samples $\tilde{x}_{ij}$, $j := 1, \ldots, M_2$, of size n_i from the null distribution.
   (b) Determine $\phi\left(\tilde{x}_{ij}\right)$ as described in section iii.1.
(ii) The probability mass function $\hat{f}_\Phi$ of Φ is approximately given by $\mathbb{P}(\Phi = p) := \frac{1}{NM_2}\left|i, j : \phi\left(\tilde{x}_{ij}\right) = p\right|$.

(iii.3) Computing the p-values for the joint hypothesis

In the previous subsection, we have shown how the distribution of p-values under the null hypothesis can be estimated if the null distribution is fully specified. We, however, need to know the distribution of the p-values if the parameters $\theta_1, \ldots, \theta_N$ are unknown. Therefore, we have to apply a second level of parametric bootstrap to test the joint hypothesis that all data come from negative binomial distributions.

Again, let $x = (x_1, \ldots, x_N)$ be our count data samples, and let $n_i := |x_i|$ and $M_3 \in \mathbb{N}_+$ be a positive integer. Furthermore, let $\Theta(x_i)$ be the estimate of the parameters θ of the null distribution based on sample x_i, and let $T(y, f)$ be a statistic suitable to test whether sample y comes from a distribution with probability mass function (pmf) f. We proceed as given below:

(i) For $i \in \{1, \ldots, N\}$:

 (a) Find an estimate $\hat{\theta}_i := \Theta(x_i)$ of the parameters of the null distribution.
 (b) Find the p-value $p_i := \phi(x_i)$ using the method from section iii.1.

(ii) With $\hat{\theta} := \left(\hat{\theta}_1, \ldots, \hat{\theta}_N\right)$ compute $\hat{f}_\Phi$ as described in section iii.2.

(iii) With $p := (p_1, \ldots, p_N)$, determine $t_0 := T(p, \hat{f}_\Phi)$.

(iv) For $j \in \{1, \ldots, M_3\}$:

 (a) For $i \in \{1, \ldots, N\}$, draw a sample $\tilde{x}_{ij}$ of size n_i from the null distribution with parameters $\hat{\theta}_i$.
 (b) Compute t_j with the steps 1-3 applied to the joint sample $\tilde{x}_j := \left(\tilde{x}_{1j}, \ldots, \tilde{x}_{Nj}\right)$.

(v) The approximate p-value for the joint hypothesis is given by the fraction of samples that had an at least equally large test statistic: $\phi(x) := \frac{1}{NM_3} \left|j : t_j \geq t_0\right|$.

(iii.4) Estimating the parameters

The procedures outlined above require parameter estimates that fit the observed data well. In this subsection, we describe how the parameters can be estimated efficiently based on sample data. However, as not all samples may contain useful information to test our base hypothesis, we start by discussing how ignoring non-informative samples could be of advantage.

Samples consisting only of zero-counts do not contain useful information on the distribution family they have been drawn from. Many distribution families have parameters that make zeros arbitrarily likely. Therefore, we would be unable to determine from which of these distributions a zero-sample has been drawn from. As a consequence, considering zero-samples could decrease the power of a test applied to check from which distribution family samples were drawn. For this reason (and to save computation time), it is beneficial to neglect samples consisting of zeros only and to focus on samples with at least one non-zero observation.

Considering only samples with at least one non-zero observation changes the hypothesized null distribution. Even if the true distribution yields zero-samples frequently, we will only consider samples with at least one non-zero observation. We therefore have to adjust our parameter estimates accordingly.

Our goal is to check whether the negative binomial distribution is appropriate to model our count data. If we disregard zero-samples, we therefore consider a negative binomial distribution conditioned such that zero-samples are impossible. In this paper, we call this distribution the "zero-sample truncated negative binomial distribution" (ZSTNB).

Besides the ZSTNB, we also regard the analogously defined "zero-sample truncated Poisson distribution" (ZSTP), which is a limiting distribution of the ZSTNB. The ZSTP is important if parameter estimates for the ZSTNB do not exist. In addition, we use the ZSTP to check the power of our approach. In this subsection, we focus on deriving estimators for the parameters of the ZSTNB and ZSTP, whereas we provide instructions on how to generate samples from these distributions in the subsection below.

There are different methods to estimate parameters based on a sample of observations. Commonly used techniques are maximum likelihood estimation and method of moment estimation [14]. While maximum likelihood estimators have favourable statistical properties and are highly efficient in general, the method of moment estimators are often easier to compute. Because our testing procedure requires us to estimate parameters an excessive number of times, we follow Famoye [13] in estimating parameters with the method of moments.

The idea behind the method of moments is to compute the moments of a distribution based on its parameters θ and equate the results with the respective sample moments. Then, the parameter estimates $\hat{\theta}$ are computed by solving this equation system. For example, consider a distribution with the parameters θ_1 and θ_2 and let μ_S and σ_S^2 be the sample mean and variance. The true mean μ and variance σ^2 of the distribution can be computed as functions of the parameters:

$$\begin{aligned} \mu &= g_\mu(\theta_1, \theta_2) \\ \sigma^2 &= g_{\sigma^2}(\theta_1, \theta_2)\,. \end{aligned} \tag{A2}$$

The method of moments parameter estimates $\hat{\theta}_1$ and $\hat{\theta}_2$ are computed by replacing μ and σ^2 on the right hand side of equation system (A2) with their respective sample equivalents μ_S and σ_S^2 and solving the system for θ_1 and θ_2.

Before we proceed, we formalize the notion of zero-sample truncated distributions.

Definition 1. Let $Y := (Y_1, \ldots, Y_n)$ be a random vector consisting of independently and identically distributed random variables. Then we say that $X := Y \big| (\exists i \in \{1, \ldots, n\} : Y_i \neq 0)$ follows a zero-sample truncated distribution.

Strictly speaking, zero-sample truncated distributions are multivariate distributions, because the individual sampling results X_i are not independent of each other. What we regarded as a sample consisting of multiple identical independent draws before turns out to be a *single* draw from a multivariate distribution. Therefore, the distribution does not have a univariate mean and variance, as would be required for the method of moments.

However, we can still apply the method of moments if we consider quantities analog to the sample mean and variance in the univariate case. Let $X := (X_1, \ldots, X_n)$ be a zero-sample truncated random variable derived from the independent random variables $Y := (Y_1, \ldots, Y_n)$ with probability mass function (pmf) f, mean μ, and variance σ^2 respectively. Our sample mean $\mu_S = \frac{1}{n} \sum_i X_i$ and sample variance $\sigma_S^2 = \frac{1}{n-1} \sum_i (X_i - \mu_S)^2$ will resemble the mean and variance of a single entry of X. Therefore, we make the following definitions:

Definition 2. We say that $\bar{f}(x_1) := \mathbb{P}(X_1 = x_1)$ is the sample pmf and $\bar{F}(x_1) := \mathbb{P}(X_1 \leq x_1)$ is the sample cmf.

Definition 3. We say that $\bar{\mu} := \mathbb{E}(X_1)$ is the expected sample mean, and $\bar{\sigma}^2 := \mathbb{V}(X_1)$ is the expected sample variance.

Note that the index "1" used above is not of importance, because the random variables $X_1, \ldots, X_n$ are identically distributed and thus exchangeable.

To ease computation of the quantities defined above, we make the following observations:

Lemma 1. *It is* $\bar{f}(x_1) = \begin{cases} \frac{f(0)-f(0)^n}{1-f(0)^n} & \textit{if } x_1 = 0 \\ \frac{f(x_1)}{1-f(0)^n} & \textit{else.} \end{cases}$

Proof. If $x_1 = 0$, then

$$\begin{aligned}
\bar{f}(0) &= \mathbb{P}(X_1 = 0) \\
&= \mathbb{P}\big(Y_1 = 0 \big| \exists i \in \{1, \ldots, n\} : Y_i \neq 0\big) \\
&= \frac{\mathbb{P}(Y_1 = 0 \wedge \exists i \in \{1, \ldots, n\} : Y_i \neq 0)}{1 - \mathbb{P}(Y = 0)} \\
&= \frac{\mathbb{P}(Y_1 = 0 \wedge \exists i \in \{2, \ldots, n\} : Y_i \neq 0)}{1 - \mathbb{P}(Y = 0)} \\
&= \frac{\mathbb{P}(Y_1 = 0) \left(1 - \mathbb{P}(Y_1 = 0)^{n-1}\right)}{1 - \mathbb{P}(Y_1 = 0)^n} \\
&= \frac{f(0) - f(0)^n}{1 - f(0)^n}.
\end{aligned}$$

Here, we used that the entries of the vector Y are identically independently distributed.

If $x_1 \neq 0$, then $\exists i \in \{1, \ldots, n\} : Y_i \neq 0$. Hence,

$$\begin{aligned}
\bar{f}(x_1) &= \mathbb{P}(X_1 = x_1) \\
&= \mathbb{P}\big(Y_1 = x_1 \big| \exists i \in \{1, \ldots, n\} : Y_i \neq 0\big) \\
&= \frac{\mathbb{P}(Y_1 = x_1 \wedge \exists i \in \{1, \ldots, n\} : Y_i \neq 0)}{1 - \mathbb{P}(Y = 0)} \\
&= \frac{\mathbb{P}(Y_1 = x_1)}{1 - \mathbb{P}(Y_1 = 0)^n} \\
&= \frac{f(x_1)}{1 - f(0)^n}.
\end{aligned}$$

This concludes the proof. □

Corollary 1. *It is* $\bar{\mu} = \frac{\mu}{1-f(0)^n}$ *and* $\bar{\sigma}^2 = \frac{\sigma^2+\mu^2}{1-f(0)^n} - \bar{\mu}^2 = \frac{\sigma^2}{1-f(0)^n} - \frac{f(0)^n\mu^2}{(1-f(0)^n)^2}$.

Proof. Direct computation yields

$$\begin{aligned}\bar{\mu} &= \mathbb{E}(X_1) \\ &= \sum_{i\in\mathbb{N}_+} i\frac{f(i)}{1-f(0)^n} \\ &= \frac{1}{1-f(0)^n}\sum_{i\in\mathbb{N}_+} if(x_1) \\ &= \frac{\mu}{1-f(0)^n}.\end{aligned}$$

Similarly, for the expected sample variance,

$$\begin{aligned}\bar{\sigma}^2 &= \mathbb{E}\left(X_1^2\right) - \bar{\mu}^2 \\ &= \sum_{i\in\mathbb{N}_+} i^2\frac{f(i)}{1-f(0)^n} - \bar{\mu}^2 \\ &= \frac{1}{1-f(0)^n}\sum_{i\in\mathbb{N}_+} i^2 f(i) - \bar{\mu}^2 \\ &= \frac{1}{1-f(0)^n}\left(\sigma^2+\mu^2\right) - \bar{\mu}^2 \\ &= \frac{\sigma^2}{1-f(0)^n} - \frac{f(0)^n\mu^2}{(1-f(0)^n)^2}.\end{aligned}$$

□

Now we can apply our general findings to find method of moments estimators for the parameters of the ZSTNB and ZSTP. For convenience, we parameterize the negative binomial distribution with the parameters r and p as described in Appendix i (equation (A16)).

We start by considering the expected sample mean of the ZSTNB. The negative binomial distribution has mean $\mu = \frac{r(1-p)}{p}$ and variance $\sigma^2 = \frac{r(1-p)}{p^2}$. Hence,

$$\begin{aligned}\bar{\mu} &= \frac{\mu}{1-f(0)^n} \\ &= \frac{r(1-p)}{p(1-p^{rn})}.\end{aligned} \tag{A3}$$

This is equivalent to

$$1 - p^{rn} = \frac{\mu}{\bar{\mu}}. \tag{A4}$$

For the expected sample variance, we get

$$\begin{aligned}\bar{\sigma}^2 &= \frac{\sigma^2+\mu^2}{1-p^{rn}} - \bar{\mu}^2 \\ \text{with (A4)} \quad &= \frac{\bar{\mu}\left(\sigma^2+\mu^2\right)}{\mu} - \bar{\mu}^2 \\ &= \bar{\mu}\frac{1+r(1-p)}{p} - \bar{\mu}^2,\end{aligned} \tag{A5}$$

which is equivalent to

$$r = \frac{p\left(\bar{\sigma}^2+\bar{\mu}^2\right) - \bar{\mu}}{\bar{\mu}(1-p)}. \tag{A6}$$

Inserting (A6) in (A3) leads after some algebra to

$$\begin{aligned} 0 &= \frac{\bar{\mu}}{p} - \bar{\mu}^2 p^{rn} - \bar{\sigma}^2 \\ \text{with (A6)} \quad &= \frac{\bar{\mu}}{p} - \bar{\mu}^2 p^{n\frac{p\left(\bar{\sigma}^2+\bar{\mu}^2\right)-\bar{\mu}}{\bar{\mu}(1-p)}} - \bar{\sigma}^2, \end{aligned} \tag{A7}$$

which can be numerically solved for p if the expected sample mean and variance $\bar{\mu}$ and $\bar{\sigma}^2$ are replaced with the observed sample mean and variance μ_S and σ_S^2.

It can be shown with basic techniques that equation (A7) has at most two zeros in the interval $(0, 1)$, one of which is $p_l := \frac{\bar{\mu}}{\bar{\sigma}^2+\bar{\mu}^2}$. However, inserting p_l in equation (A6) would lead to an r-estimate of 0. We know that $r > 0$. Therefore, p_l cannot be a valid parameter estimate. Because $r > 0$, we also know that the true estimate $\hat{p}$ must be larger than p_l. We thus can use a simple bisection method to find $\hat{p}$ in the interval $(p_l, 1)$. After computing $\hat{p}$ by this means, we insert $\hat{p}$ into equation (A6) to get our estimate $\hat{r}$ for the parameter r.

It is possible that the method of moments estimator $\hat{p}$ does not exist. This happens if equation (A7) does not have a root in $(p_l, 1)$, which is the case if, and only if,

$$0 > \bar{\mu} - \bar{\mu}^2 e^{-n\left(\frac{\bar{\sigma}^2}{\bar{\mu}}+\bar{\mu}-1\right)} - \bar{\sigma}^2. \tag{A8}$$

If this happens, we will assume that the sample came from the ZSTP, which is a limiting case of the ZSTNB. As we will see below, the methods of moments estimator exists for the ZSTP in most instances.

We proceed by deriving an estimator for the parameter of the ZSTP. Oftentimes, the Poisson distribution is directly parameterized by its mean μ. The expected sample mean is given by

$$\begin{aligned} \bar{\mu} &= \frac{\mu}{1 - f(0)^n} \\ &= \frac{\mu}{1 - e^{-n\mu}}, \end{aligned} \tag{A9}$$

which is equivalent to

$$\mu = \frac{1}{n} \mathrm{W}\left(-n\bar{\mu}e^{-n\bar{\mu}}\right) + \bar{\mu}. \tag{A10}$$

Here, W denotes the Lambert W-function, which is the inverse function of $h(W) := We^W$. Packages with efficient implementations of the Lambert W-function exist for many programming languages. This makes it easy to compute the parameter estimate for μ.

The right hand side of equation (A10) assumes a real value if $\bar{\mu} > \frac{1}{n}$. However, if, and only if, there is exactly one non-zero count value in the sample and this count value is 1, then $\bar{\mu} = \frac{1}{n}$. In this case, the parameter estimate does not exist, because the sampling result could be made arbitrarily likely by choosing a very small value for μ. Therefore, we adjust the procedures outlined in the sections above so that samples with $\bar{\mu} = \frac{1}{n}$ always lead to p-values of 1. Furthermore, we say that the distribution of p-values based on a null distribution whose parameters were estimated based on such a sample returns 1 with probability 1.

(iii.5) Generating random numbers

In this subsection, we describe algorithms to draw random numbers $(x_1, \ldots, x_n)$ form the ZSTNB and the ZSTP. Drawing numbers from these distributions is a crucial component of the algorithms described in the subsections above. Though efficient random number generators are available for the negative binomial distribution and the Poisson distribution, drawing numbers from zero-sample truncated distributions is a more complicated task. However, with a combination of the algorithms given below, samples can be generated with almost the same efficiency as samples from the “classical” negative binomial and Poisson distribution.

The naive approach to drawing samples from zero-sample truncated distributions is to generate samples from the original distribution until a sample with at least one non-zero entry is obtained. This approach is very efficient if the probability that the sample consists of zeros only is small. If f is the pmf of the original function and n is the sample size, then $f(0)^n$ is the probability that the sample consists of zeros only. If this quantity is small, only few samples have to be generated until a suitable one is found. Therefore, the alternative approaches below should be applied only if $f(0)^n$ is large.

To avoid an excessive number of trials until a suitable sample is found, we propose to first draw the sum $x_\Sigma := \sum_{i=1}^n x_i$ of all entries of the sample and to determine the values of the summands afterwards.

Recall that $x_\Sigma \neq 0$ for zero-sample truncated distributions. For both the negative binomial and the Poisson distribution, the sum of n independent and identical trials is known to be negative binomially and Poisson distributed as well. Hence, the distribution of x_Σ, which is constrained to be positive, is easy to derive. If f_Σ is the pmf of the random variable Y_Σ modelling the sum of n independent draws from the original distribution and X_Σ is the random variable from which x_Σ is drawn, then for $x_\Sigma \neq 0$,

$$\begin{aligned}\mathbb{P}(X_\Sigma = x_\Sigma) &= \mathbb{P}\big(Y_\Sigma = x_\Sigma \big| Y_\Sigma \neq 0\big) \\ &= \frac{f_\Sigma(x_\Sigma)}{f_\Sigma(0)}. \end{aligned} \tag{A11}$$

If $f(0)^n$ is large, $\frac{f_\Sigma(x_\Sigma)}{f_\Sigma(0)}$ is usually small, unless x_Σ is small. We therefore suggest the following procedure:

(i) Compute a high quantile x_{max}, e.g. the $q = 0.99999$ quantile, of Y_Σ.
(ii) For $1 \leq x_\Sigma \leq x_{\text{max}}$, compute $\mathbb{P}(X_\Sigma = x_\Sigma)$.
(iii) Draw an integer x_Σ, $1 \leq x_\Sigma \leq x_{\text{max}}$, according to the probabilities computed above.

Using x_{max} as upper bound for x_Σ introduces a potential error, because for both the negative binomial and the Poisson distribution arbitrarily high values occur with a positive probability. However, bounding x_Σ makes it easy to apply common random number generators to draw from a zero-truncated distribution. If a hard boundary for the error introduced by using a finite x_{max} is desired, the quantile q can be chosen as $q = f(0) + (1 - \epsilon)\,(1 - f(0))$. Then, a value larger than x_{max} occurs only with probability ϵ.

After drawing the sum x_Σ, we need to determine the individual count values x_i. For small values of x_Σ, only few different configurations of count values are possible. Each of these configurations has a probability, which can be computed easily. Then, the final configuration can be drawn according to these probabilities. For large values of x_Σ, we propose to use a Metropolis-Hastings algorithm to determine the final configuration. We provide details below.

If $x_\Sigma = 1$, we can just set $x_1 := 1$ and $x_i := 0$ for $2 \leq i \leq n$. The order of the sample does not matter in this paper. Therefore, it is appropriate to set the first entry to 1 always. If, for a different application, the order of the entries is important, a random shuffling algorithm can be applied to make the ordering unbiased.

If $x_\Sigma = 2$, there are two possible configurations: 2 entries of 1 or 1 entry of 2 while all remaining entries of the sample are 0, respectively. The probabilities for these configurations are easy to compute. Since the computations are simple but tedious, we do not present them here. After the probabilities of the configurations have been determined, the configuration of the sample is drawn randomly according to the probabilities.

If $x_\Sigma = 3$, the number of possible configurations is still small and the respective probabilities are easy to compute explicitly. As above, we do not present the computations here. The final configuration is then drawn according to the computed probabilities.

As $x_\Sigma \geq 4$ becomes large, the number of possible configurations increases quickly. In practice it happens rarely that $x_\Sigma \geq 4$ if $f(0)^n$ is large. In fact, often $x_{\text{max}} < 4$. Nonetheless, dependent on when $f(0)^n$ is considered large, it can indeed happen that $x_\Sigma \geq 4$. In this case, we propose to use a Metropolis-Hastings algorithm to determine the configuration of the sample. This algorithm accepts and rejects changes to a given distribution based on the likelihood ratio of the original and new sample. The algorithm is as follows:

(i) Set $x := (x_1, \ldots, x_n)$ to some arbitrary initial condition with $\sum_{i=1}^n x_i = x_\Sigma$.
(ii) Randomly draw two distinct indices i, j with $1 \leq i, j, \leq n$, $x_i \neq 0$, and $x_i \neq x_j$.
(iii) Create a copy x' of x and set $x'_i := x_i - 1$ and $x'_j := x_j + 1$.
(iv) Determine $\mathbb{P}(x')$ and $\mathbb{P}(x)$.
(v) If $\mathbb{P}(x') \geq \mathbb{P}(x)$ set $x := x'$. Otherwise, set $x := x'$ with probability $\frac{\mathbb{P}(x')}{\mathbb{P}(x)}$.
(vi) Repeat steps 2 to 5 a large number of times.

If a sample from the ZSTP distribution shall be drawn, the process can be replaced by a simple draw from a multinomial distribution with n bins and uniform probabilities $\frac{1}{n}$.

(iii.6) Reusing partial results

The approach outlined above requires us to draw $M_1M_2M_3$ samples for each count sample x_i, $i = 1, \ldots, N$, and to determine parameter estimates and evaluate the Anderson-Darling statistic for each of these samples.

Hence, the nested parametric bootstrap method is computationally costly. However, computations can be sped up if earlier results are reused.

As the distribution for the samples $x_i = (x_{i1}, \ldots, x_{in_i})$ is permutation-invariant, the only information that we use is how often each possible count value occurred. That is, if $\nu_{ik} := \left|j : x_{ij} = k\right|$, then a set $\nu_i := \left\{(k, \nu_{ik}) \,\middle|\, \nu_{ik} \neq 0\right\}$ containing all non-zero ν_{ik} suffices to describe x_i. Furthermore, each sample x_i has a specific parameter estimate $\Theta(x_i)$, statistic value $T(x_i, \Theta(x_i))$, and p-value $\phi(x_i)$ associated to it. Therefore, it is sufficient to compute $\Theta(x_i)$, $T(x_i, \Theta(x_i))$, $\phi(x_i)$ for each ν_i only once. This can be implemented efficiently via hash-maps with hashes of ν_{ik} as keys.

Dependent on which partial results are reused, reusing results can lead to precision loss of the overall algorithm. The quantities $\Theta(x_i)$ and $T(x_i, \Theta(x_i))$ are computed with deterministic algorithms. Therefore, reusing these quantities comes with no additional cost. The p-values $\phi(x_i)$, however, are computed with a parametric bootstrap technique. Therefore, reusing these results can lead to an increased variance of the results. Nonetheless, the performance gain obtained from reusing partial results usually outweighs the precision loss. In fact, since p-values do not have to be computed as frequently if results are reused, a large value M_1 can be chosen with minor increase in computation time. This usually leads to more precise results in the end.

(iii.7) Determining the accuracy of the approach

The nested bootstrap method for testing the distribution of count data is based on frequent resampling and therefore subject to error. The p-value resulting from the nested bootstrapping is a random variable. The variance of the result can be arbitrarily decreased by choosing large sample numbers M_1, M_2, and M_3. Nonetheless, it would be desirable to get an estimate of the error. We therefore suggest to repeat the procedure M_4 times and to determine the standard deviation of the resulting p-values as measure for the error.

Repeating the procedure also decreases the error further, as the resulting mean value will be close to the actual p-value than each result individually. Since the nested bootstrap method is computationally expensive, we chose a moderate $M_4 = 20$ in this paper.

(iv) Check for model bias

We tested our model for bias with an observed versus predicted regression as described by Haefner [15]. If the model is accurate, predictions should be close to the observed data. Hence, all data should be close to a line with slope 1 and intercept 0, when observed data are plotted against predictions. The test described by Haefner [15] checks the null hypothesis “slope = 1 and intercept = 0”. If the model is unbiased, the resulting p-value should be high so that the null hypothesis cannot be rejected.

The test requires that all predictions follow normal distributions with similar variances. Therefore, a transformation step was required to make the test applicable to our model. To obtain normally distributed predictions, we considered sums of identically and independently distributed (i.i.d.) random variables. These sums are approximately normally distributed according to the central limit theorem. We generated the sets of i.i.d. random variables by considering the homogenized count data obtained as described in section i. We considered the total number of boaters observed in each shift. Then we proceeded as follows:

(i) Using our fitted model and knowing the sample sizes at each survey location, we computed the predicted standard deviation of the count data for each survey location.
(ii) We normalized the count data so that they had a predicted standard deviation of 1.
(iii) We normalized our model predictions accordingly.
(iv) We applied the method by Haefner [15] to the normalized observations and predictions and computed the p-value.

We applied the method described above to a validation data set distinct from the data set used to fit the model. To generate the validation data set, we randomly selected 30% of all survey shifts. The rest of the data were used to fit the model.

(v) Accuracy of the predicted mean boater flow

In this section, we describe the method we applied to assess the accuracy of our model's predictions. A commonly used measure for model accuracy is the coefficient of determination R^2. However, R^2 is not

directly applicable in our case, because we assume that the variance of our count data increases proportional to the respective mean values. That is, R^2 would put higher emphasis on large count data than desired. Furthermore, R^2 would provide a measure for the "absolute" error, while the relative error is often of higher interest to managers. Considering the relative error, in turn, is difficult if the data are dominated by low counts.

To apply R^2 anyway, we normalized our count data as described in section iv. The resulting data are approximately normally distributed around the predicted mean values with a uniform standard deviation of 1. As a result, a meaningful R^2 value can be computed.

The procedure outlined above exploits that we can extract identically distributed traffic observations for each survey location. This is not easily possible for individual origins and destinations, because boaters from any origin travelling to any destination could be observed at any location. Since the survey location affects the distribution of observed boaters, the only way to obtain identically distributed observations for individual origins and destinations would be to focus on data collected at a specific set of simultaneously operated survey locations. This constraint, however, would require us to neglect large portions of our data set. Therefore, we refrained from applying a normalization procedure and computing R^2 values for these model predictions. Instead, we conducted a graphical comparison of predicted and observed count values.

We determined the predicted and observed count values based on our survey set up. That is, our predictions took into account where and when we conducted surveys. Then we plotted the observed count values against predicted mean values. Since the model is stochastic, we expect the observed values to deviate from the predictions. Nonetheless, the predicted-observed pairs can be expected to be close to a line with slope 1 and intercept 0 if the model is accurate. To quantify the deviations between the predictions and observations, we computed the mean absolute error between expected and observed count values relative to the predicted standard deviations.

To identify strengths and weaknesses of our model, we conducted the analyses from four perspectives:

(i) To assess the model's ability to predict the flow between individual origin-destination pairs, we plotted for each donor-recipient pair the number of observed and predicted boaters.
(ii) To assess the model's ability to determine the repulsiveness of donor jurisdictions, we plotted observed and predicted boaters for each individual donor jurisdiction.
(iii) To assess the model's ability to estimate the attractiveness of recipient lakes, we plotted observed and predicted boaters for each recipient lake.
(iv) To assess the model's ability to predict the boater flow along roads, we plotted observed and predicted boaters for each survey location.

As the classical R^2 value was inapplicable for a large portion of our results and measures the model's accuracy in predicting a specific quantity of interest only, we also computed the pseudo R^2 value suggested by Nagelkerke [16]. We outlined our approach in the main text.

Similar to the check for model bias, we applied the checks for model accuracy to a validation data set distinct from the data set used to fit the model. We tested model accuracy based on the same validation set used to check the model for an overall bias.

(b) Results

(i) Shape of the temporal traffic pattern

To check whether the von Mises distribution is appropriate to model the temporal traffic pattern, we compared the fitted von Mises distribution to step function distributions fitted to the data. In Figure A2, it is visible that the distributions resemble each other in shape. A different version of this figure can be found in the main text (Figure 4). Besides a graphical comparison, we also compared the distributions based on the model selection criterion AIC. The AIC values of the distributions were close, though the best step function model ($n = 10$) was slightly lower than the von Mises distribution ($\Delta\text{AIC} = 2.9$).

(ii) Distribution of count data

We tested whether our count data came from a negative binomial distribution. We obtained a p-value of 0.27 with standard deviation 0.05. To test the power of our approach, we also applied the nested parametric bootstrap method to test whether the count data are Poisson distributed. This hypothesis resulted in a p-value of 0.

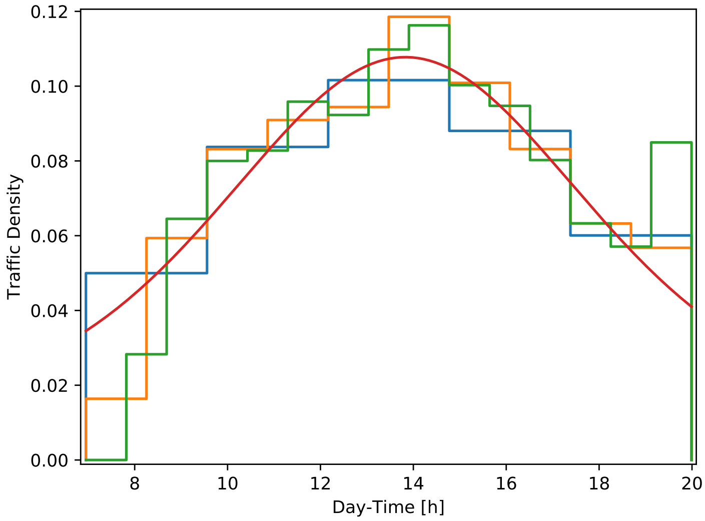

Figure A2: Comparison of different step-function distributions with the von Mises distribution. The curves depict the probability density functions of the best-fit step distributions with $n = 5$ intervals (blue), $n = 10$ intervals (orange), $n = 15$ intervals (green), and the von Mises distribution (red). To ease comparison, all curves were normalized to be probability distributions on the time interval 7AM till 8PM, for which we have count data. It is visible that the shapes of the step functions resemble the shape of the von Mises distribution.

(iii) Check for model bias

The check for model bias resulted in a p-value of 0.22.

(iv) Accuracy of the predicted mean boater flow

The observed versus predicted analysis with normalized count data for each inspection station is presented in the main text (Figure 8). We obtained R^2 values of 0.79 for the fitting data set and 0.73 for the validation data.

The observed versus predicted plots with non-normalized data are displayed in Figure A3. It is visible that our model has difficulties to predict the number of travelling boaters for separate origin-destination combinations with significant traffic (Figure A3a). There are several jurisdiction-lake pairs for which the observed value is far from the mean of the estimated distribution. The same applies to the plot displaying the model's ability to predict the total inflow to lakes (Figure A3d). However, the predicted and observed values match relatively well for the total outflow of jurisdictions (Figure A3c) and the flow through the survey locations (Figure A3b).

Unlike suggested by the visual appearance, the mean absolute relative errors were lowest for the traveller counts for individual origin-destination pairs and individual destinations. The corresponding error values were 0.008 standard deviations and 0.11 standard deviations, respectively, for the validation data. In contrast, the mean errors for the traffic observed at survey locations and the traffic from individual origins were 2.06 and 1.11 standard deviations, respectively. Note, however, that the error for origin-destination pairs and destinations increased to 1.59 and 1.75 standard deviations when origin-destination pairs and destinations with expected

(c) Discussion

(i) Methods

Before discussing the main validation results, we discuss the model validation methods that we applied.

We used a graphical comparison method to check whether the von Mises distribution is appropriate to model the temporal variations of traffic. Of course, a more rigorous statistical test, e.g. the Anderson-Darling

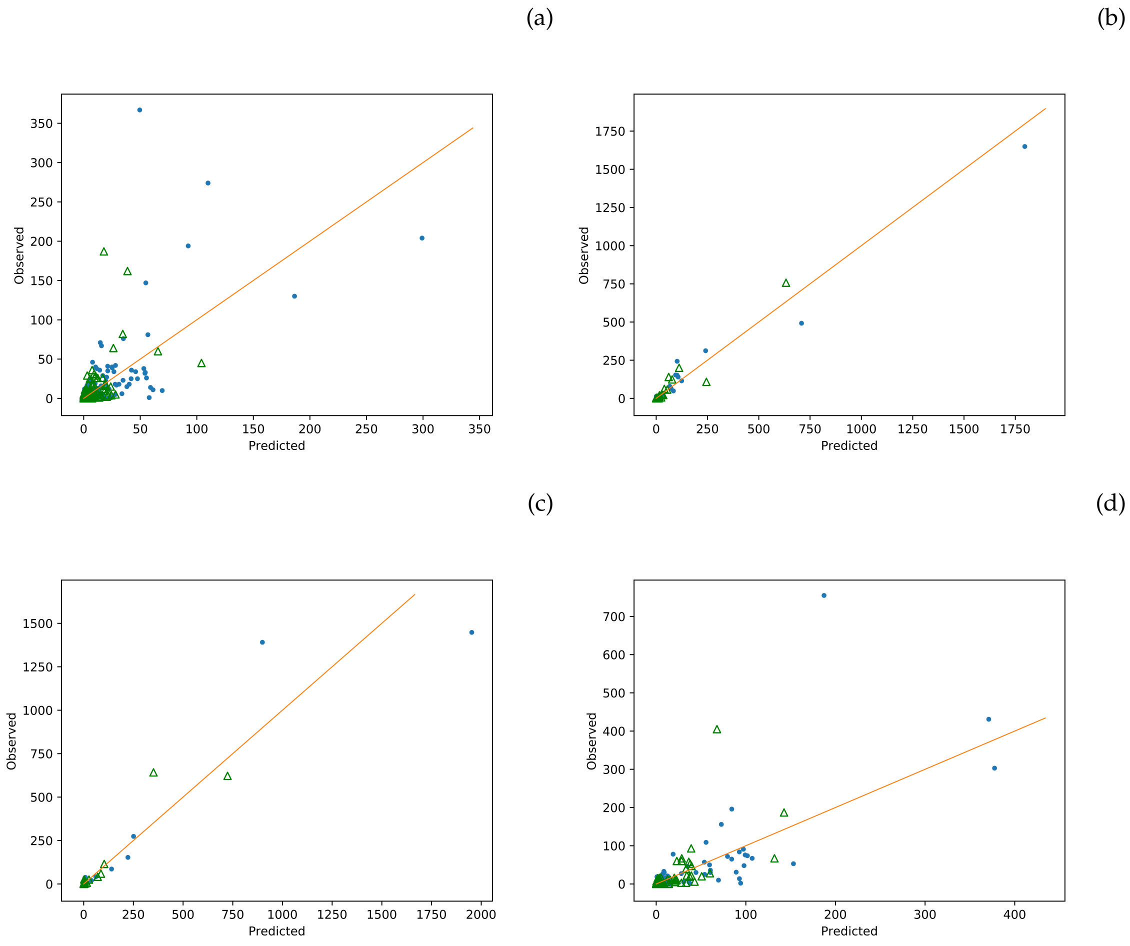

Figure A3: Observed versus predicted count values. The markers depict the the predicted mean and the observed count value of boaters for (a) each jurisdiction-lake pair, (b) each survey location, (c) each source jurisdiction, and (d) each recipient lake. If the model were perfect, all points would be close to the solid line, at which predicted mean and observed value are equal. The blue dots correspond to the data used to fit the model, whereas the green hollow triangles correspond to the validation data. The mean relative errors in units of standard deviations are as follows (validation data / fitting data): (a) 0.008 / 0.012; (b) 2.06 / 2.91; (c) 1.11 / 1.58; (d) 0.11 / 0.16.

test, would have been possible, too. However, since such tests require identically distributed samples in general, we would not have been able to use all available data for these tests. Furthermore, statistical tests may be suitable to show that a hypothesis is wrong, but other methods may be more appropriate to confirm a null hypothesis. The observation that distributions without pre-imposed shape mimic the von Mises distribution is a strong hint suggesting that the von Mises distribution is appropriate to model temporal traffic variations.

Our nested parametric bootstrap method for testing whether the count data come from a negative binomial distribution is computationally expensive and can lead to imprecise results. However, in simulations (not shown here) the method proved to be powerful in discerning negative binomially distributed data from data coming from other distributions. This observation goes in line with the low p-value with which the nested parametric bootstrap showed that the count data did not come from a Poisson distribution. Though the computational constraints make it impossible to generate a large number of bootstrap samples, the error of the method was sufficiently small to allow well-informed inference.

The observed versus predicted analysis that we used to check for model bias is a suitable method to confirm that the model is implemented correctly. However, though the method is able to identify an overall bias in our predictions, the method would be unable to identify biases in subsets of our data. For example,

if our model would underestimate the traffic to attractive lakes and overestimate the traffic to unattractive lakes, the aggregate predictions would not show a bias. Therefore, the method cannot be used to measure the accuracy of our predictions.

The graphical observed versus predicted analysis we conducted to assess the accuracy of our model is a suitable tool to measure model performance, as it is easy to check which parts of the model fit the data well and where inaccuracies result from. The mean relative absolute error is a valuable quantitative measure to put depicted inaccuracies into context. However, since the mean absolute relative error is an aggregated metric, it can be dominated by quantities that are easy to predict but of minor interest when evaluating model accuracy. For example, 99% of the origin-destination pairs considered in our study were estimated to have very little boater traffic, corresponding to 2.1% of all boater observations in total. Though our model successfully identified low-traffic origin-destination pairs, it was less successful in determining how many boaters travel between the origins and destinations that *do* have significant traffic. This shortcoming is not visible if alone the aggregated error is considered. Therefore, visual inspection of observed versus predicted plots yields more insights into model accuracy than considering the mean relative absolute error.

As an alternative to a graphical analysis, a nested bootstrap method could be applied to check whether a statistic applied to the observed data would be likely to return the observed value if the model is correct. However, given the apparent inaccuracies, which far exceed expected standard deviations, it is not necessary to apply additional tests to confirm that the model is inaccurate. Therefore, we abstained from implementing this computationally expensive validation method.

(ii) Results

We have checked two main hypotheses our model is based on and validated the accuracy of the model's predictions. Overall, our test results indicate that the model assumptions are appropriate. However, the model's predictions turned out to suffer from inaccuracies.

For the temporal traffic pattern model, the fitted step function distributions resembled the von Mises distribution and resulted in only slightly better AIC values. This justifies the choice of the von Mises distribution to model the temporal traffic pattern, also considering that (1) the von Mises distribution has a lower risk of being overfitted to the data, and (2) the von Mises distribution provides reasonable estimates for night-time traffic, for which we have no data. Hence, it is appropriate to model the temporal traffic pattern with the von Mises distribution.

For the distribution of the count data, we obtained a relatively high p-value for the null hypothesis that our data are negative binomially distributed. Even though this does not prove that the data are negative binomially distributed, this result does not allow us to conclude the opposite. Since a distribution test with the null hypothesis that the data are Poisson distributed resulted in a very small p-value, our test appears to be sufficiently powerful to reject wrong hypotheses. This supports the negative binomial hypothesis further. Hence, the negative binomial distribution seems appropriate to model our count data.

Our test for an overall model bias resulted in a moderate p-value. Hence, the null hypothesis that the model yields unbiased results cannot be rejected. Therefore, we have no reason to believe that the model predictions are subject to an overall bias.

The R^2 values for the model accuracy were discussed in the main text. Our comparison of predicted and observed values has shown that our model suffers from inaccuracies. As we conducted separate checks for the model's ability to predict the outflow of donor jurisdictions and the inflow to recipient lakes, we can make informed guesses about which model component is responsible for the errors. Both the temporal pattern model and the route choice model are likely to affect all predictions similarly strongly. If these model components were the main cause for the inaccuracies, we would see the same level of inaccuracy on all predicted versus observed plots. However, we observed that our model's predictions of the outflow from jurisdictions were much more accurate that the predictions of the inflow to jurisdiction lakes (compare Figures A3c and A3d). Therefore, it is likely that our model's inaccuracies result from its inability to precisely estimate the attractiveness of lakes rather than from other model components.

The two major outliers in Figure A3c correspond to counts of boaters coming from the northern subsections of Alberta, a neighbouring province of BC. These outliers may be partially caused by difficulties to determine the origin of boaters on a sub-provincial scale. Similarly, the major outliers in Figure A3d correspond to counts of boaters travelling to subdivided large lakes. As determining the correct destination lake section from the survey data was difficult at times, more accurate results might be obtained if boaters

are asked to provide their specific destination lake access points rather than only the destination lake and a close-by city.

The mean absolute relative error between observations and predictions was low for the predictions of traffic between individual origin-destination pairs and to individual destinations (Figures A3a and A3d). This indicates that the model predicts the corresponding boater traffic accurately. Note, however, that this result is driven by the model's success in determining where boaters do *not* travel, since only few lakes and origin-destination pairs encounter significant boater traffic. In fact, the aggregate errors increased strongly when destinations and origin-destination pairs with low traffic were excluded from the analysis. However, though highly accurate traffic estimates for heavily frequented lakes would be desirable, knowing the large set of low-traffic lakes is of benefit to mangers as well.

All in all, we can conclude from the model validation results above that a more accurate model would require a more sophisticated submodel for lake attractiveness. Improving the other model components may also enhance the model accuracy but presumably not to the same extent as an improved gravity model. A more sophisticated model for lake attractiveness, however, would likely also require more covariates to distinguish between attractive unattractive lakes. This is a constraint that all models for agent traffic would face. Therefore, the limited accuracy of our model does not generally outweigh the methodological advancements of this study.

G. Identifiability of the parameters β_{lpop} and lpop_0

The confidence intervals for the parameters β_{lpop} and lpop_0 given in Table 1 in the main text are very large. That is, the correct values of these parameters are not estimable with the data that we used to fit the model. Often, such estimability issues decrease the credibility of inference and predictions drawn from a model. However, we argue that though the parameter values appear to be not estimable, our model and resulting predictions are reliable.

In Figure A4, we have plotted the contribution $f(x) := \beta_{\text{lpop}} \left(\frac{x}{x + \text{lpop}_0} \right)$ of the covariate "population in a $5\,\text{km}$ range of a lake" (here denoted x) to the lake attractiveness for two extreme parameter choices. It is visible that the contribution curves differ by no more than factor 1.5. The difference is maximal for lakes with a high surrounding population. Note that only two of the considered lakes have a surrounding population exceeding $250,000$. These lakes have a small area and therefore do not attract many boaters. Hence, these lakes do not contribute to the results significantly. For lakes with surrounding population counts below $250,000$, in turn, the estimates differ by no more than 15%. For the middle section of Okanagan Lake, the only lake section that has both a high surrounding population count and a large size, the contribution of the surrounding population count to the lake attractiveness differs by less than 5% between the models.

Note that the large range of permissible parameter values suggest that even a model without the parameter lpop_0 could fit the data well. Indeed, the AIC difference between the models with and without this parameter is less than 2, so that both models can be considered well-fitting. Nonetheless, the model with the additional parameter has the minimal AIC value and was thus chosen.

Though the discussed estimability issue does not compromise the validity of the presented estimates of boater traffic to BC, the estimates may be come inaccurate if the fitted model is applied to regions with many lakes in highly populated regions. Since such lakes are rare in BC, our model for the attractiveness of these lakes is based on a small data set. Therefore, the model should be refitted before it is applied to systems with denser population around lakes.

References

1. Fischer SM. 2019 Locally optimal routes for route choice sets. *arXiv e-prints* pp. 1–40. arXiv:1909.08801.
2. Villa ER, Escobar LA. 2006 Using moment generating functions to derive mixture distributions. *The American Statistician* **60**, 75–80.
3. Jones E, Oliphant T, Peterson P. 2001 SciPy: open source scientific tools for Python. Retrieved from https://scipy.org/.
4. Storn R, Price K. 1997 Differential evolution–a simple and efficient heuristic for global optimization over continuous spaces. *Journal of global optimization* **11**, 341–359.
5. Byrd RH, Lu P, Nocedal J, Zhu C. 1995 A limited memory algorithm for bound constrained optimization. *SIAM Journal on Scientific Computing* **16**, 1190–1208.
6. Kraft D. 1988 A software package for sequential quadratic programming. Technical Report DFVLR-FB 88-28 DLR German Aerospace Center – Institute for Flight Mechanics Köln, Germany.

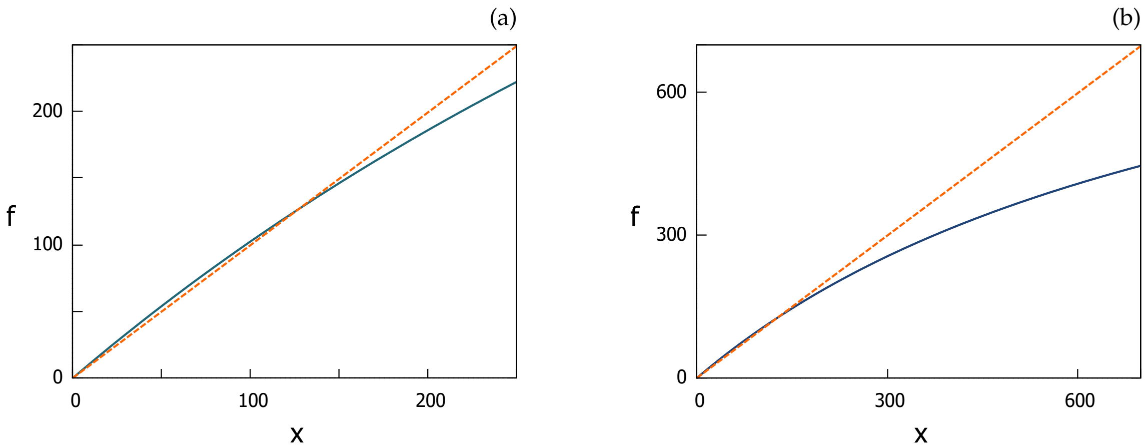

Figure A4: Contribution of the covariate "population in a 5 km range of a lake" (in thousand; denoted x) to the lake attractiveness for two extreme parameter choices. The two functions differ moderately for large population counts. The left panel shows a subsection of the right panel. Parameters: solid blue: $\beta_{\mathrm{lpop}} = 1011$, $\mathrm{lpop}_0 = 887$; dashed orange: $\mathrm{lpop}_0 = 1.182\mathrm{e}10$, $\alpha_{\mathrm{lpop}} = 1.186\mathrm{e}10$.

7. Nocedal J, Wright SJ. 2006 *Numerical optimization*. Springer series in operations research. New York: Springer 2nd edition.
8. Aho K, Derryberry D, Peterson T. 2014 Model selection for ecologists: the worldviews of AIC and BIC. *Ecology* **95**, 631–636.
9. Akaike H. 1974 A new look at the statistical model identification. *IEEE Transactions on Automatic Control* **19**, 716–723.
10. Schwarz G. 1978 Estimating the dimension of a model. *The Annals of Statistics* **6**, 461–464.
11. Varin C, Vidoni P. 2005 A note on composite likelihood inference and model selection. *Biometrika* **92**, 519–528.
12. Varin C. 2008 On composite marginal likelihoods. *AStA Advances in Statistical Analysis* **92**, 1–28.
13. Famoye F. 1998 Bootstrap based tests for generalized negative binomial distribution. *Computing* **61**, 359–369.
14. Casella G, Berger RL. 2002 *Statistical inference*. Pacific Grove, CA: Thomson Learning 2nd edition.
15. Haefner JW. 2005 *Modeling biological systems: principles and applications*. New York: Springer 2nd edition.
16. Nagelkerke NJD. 1991 A note on a general definition of the coefficient of determination. *Biometrika* **78**, 691–692.
